# Amylopectin Chain Length Dynamics and Activity Signatures of Key Carbon Metabolic Enzymes Highlight Early Maturation as Culprit for Yield Reduction of Barley Endosperm Starch after Heat Stress

**DOI:** 10.1093/pcp/pcz155

**Published:** 2019-08-09

**Authors:** Jose Antonio Cuesta-Seijo, Alice Jara De Porcellinis, Angela H�rdum Valente, Alexander Striebeck, Cynthia Voss, Lucia Marri, Andreas Hansson, Anita M Jansson, Malene Hessellund Dinesen, Jonatan Ulrik Fangel, Jesper Harholt, Milan Popovic, Mercedes Thieme, Anton Hochmuth, Samuel C Zeeman, Teis N�rgaard Mikkelsen, Rikke Bagger J�rgensen, Thomas Georg Roitsch, Birger Lindberg M�ller, Ilka Braumann

**Affiliations:** 1 Carlsberg Research Laboratory, J.C, Jacobsens Gade 4, 1799 Copenhagen V, Denmark; 2 Department of Plant and Environmental Sciences, Copenhagen Plant Science Centre, University of Copenhagen, Hojbakkegard Alle, 2630 Taastrup, Denmark; 3 Institute of Molecular Plant Biology, ETH Zurich, Zurich 8092, Switzerland; 4 Atmospheric Environment, DTU Environmental engineering, Technical University of Denmark, Building 115, 2800 Kgs, Lyngby, Denmark; 5 Plant Biochemistry Laboratory, Department of Plant and Environmental Sciences, University of Copenhagen, 1871 Frederiksberg, Denmark

**Keywords:** Amylopectin, Barley, Cell wall invertase, Grain filling, Heat stress, Starch

## Abstract

Abiotic environmental stresses have a negative impact on the yield and quality of crops. Understanding these stresses is an essential enabler for mitigating breeding strategies and it becomes more important as the frequency of extreme weather conditions increases due to climate change. This study analyses the response of barley (*Hordeum vulgare* L.) to a heat wave during grain filling in three distinct stages: the heat wave itself, the return to a normal temperature regime, and the process of maturation and desiccation. The properties and structure of the starch produced were followed throughout the maturational stages. Furthermore, the key enzymes involved in the carbohydrate supply to the grain were monitored. We observed differences in starch structure with well-separated effects because of heat stress and during senescence. Heat stress produced marked effects on sucrolytic enzymes in source and sink tissues. Early cessation of plant development as an indirect consequence of the heat wave was identified as the major contributor to final yield loss from the stress, highlighting the importance for functional stay-green traits for the development of heat-resistant cereals.

## Introduction

Crop plants as sessile organisms are strongly influenced by their immediate environment. Adverse environmental conditions, such as salinity, drought, cold or heat, can have a negative impact on crop performance and harvestable yield (Bita and Gerats 2013). Consequently, breeding novel crop varieties designed to provide stable yields under challenging environmental conditions is a central target in current plant breeding efforts (Tester and Langridge 2010). However, yield is a complex trait determined by several complementing elements of plant development (Sreenivasulu and Schnurbusch 2012). Therefore, abiotic stress impacts plants differently depending on the developmental stage it occurs. This study is concerned with environmental stress—specifically heat—during cereal grain development, which has a negative effect on the accumulation of starch (Thitisaksakul et�al. 2012). Starch is the polysaccharide most commonly found in plants to store photosynthetic carbon-assimilates for later consumption—either as transitory starch in photosynthetic tissues or as energy reserve for the succeeding generation in specialized non-photosynthetic organs, such as cereal grains (Streb and Zeeman 2012). The major cereal crops contain up to 70% (w/w) of starch in their grains (Thitisaksakul et�al. 2012), which illustrates that efficient starch accumulation is a key contributor to cereal crop yield.

Recently, several studies investigating the impact of abiotic stress during cereal grain development, the phase of starch accumulation, focus on transcriptomics (Yamakawa et�al. 2007, Luo et�al. 2010, Sz�cs et�al. 2010, Mangelsen et�al. 2011, Goswami et�al. 2016) or proteomics (Jiang et�al. 2012, Shi et�al. 2013, Liao et�al. 2014, Kaneko et�al. 2016, Timabud et�al. 2016, Zhang et�al. 2017), while the effect on the major metabolic product of the cereal grain, starch, is not addressed. However, elevated temperatures during grain filling can limit starch accumulation and impact the molecular properties of starch resulting in reduced grain quality, e.g. increased starch gelatinization temperature (GT; Asaoka et�al. 1985, Tester et�al. 1991, Myll�rinen et�al. 1998, Umemoto et�al. 1999, Lu et�al. 2013, Lu et al. 2014, Chun et�al. 2015, Zhang et�al. 2016). The GT is the temperature at which the internal structure of starch is disrupted (Ratnayake and Jackson 2008). Gelatinization makes the starch accessible for human consumption through cooking or for other applications such as mashing during the brewing process.

Starch biosynthesis is complex process that requires multiple enzymes. Enzymes are either directly involved in amylose and amylopectin biosynthesis or in the metabolism of carbohydrates imported into grains from photosynthetic tissues, which provide the starting material for starch formation (Fig.�1). Briefly, carbohydrates from source tissues are supplied in the form of sucrose (Suc). Upon delivery to sink tissues, such as the cereal endosperm, the part of the cereal grain, where starch is stored, Suc is metabolized either by invertase (INV) or sucrose synthase (SuS). INVs hydrolyze Suc into glucose (Glc) and fructose (Fru) and depending on their cellular location they are classified as cell wall invertase (CWIN), vacuolar invertase (VIN) or cytoplasmic invertase (CIN) (Roitsch and Gonz�lez 2004). By contrast, SuS converts Suc to Fru and UDP-glucose (UDP-glc) in the presence of UDP (Ruan 2014). In addition to starch biosynthesis, Suc is further the base for other essential biochemical processes in sink tissues such as respiration and cell wall biosynthesis (Sturm and Tang 1999).

**Fig. 1:**
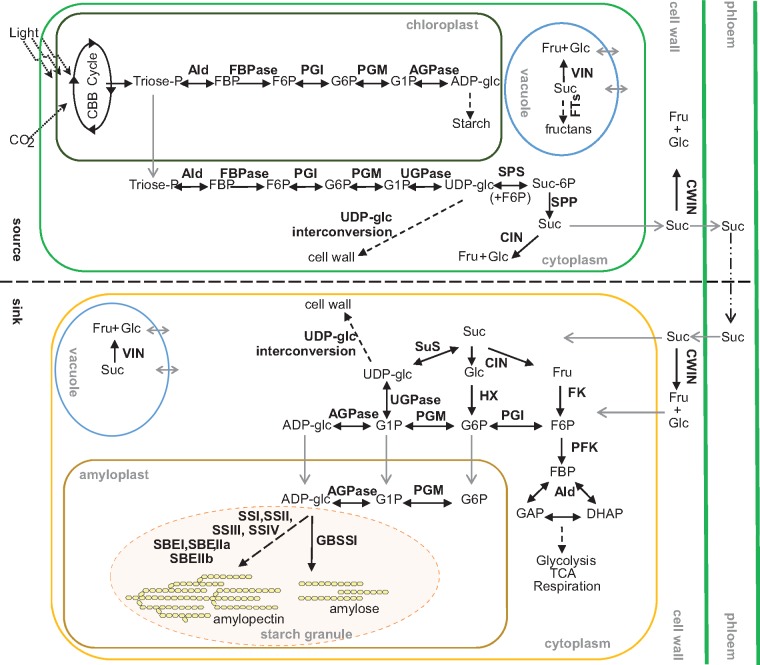
Overview on the carbon metabolism in source (leaf) and sink (grain) tissue. Photosynthetic source tissues, like barley flag leaves, assimilate carbohydrates. These are exported in the form of Suc, which is transported into sink tissue like the cereal endosperm. There, Suc is channeled into starch and cell wall biosynthesis as well as glycolysis. CBB cycle, Calvin–Benson–Bassham cycle; TCA, tricarboxylic acid cycle; concerning a comprehensive listing of abbreviations used for enzymes and metabolites, the reader is referred to the abbreviations listed below. ADP-glc, ADP-glucose; AGPase, ADP-glucose pyrophosphorylase; Ald, fructose-bisphosphate aldolase; cv., cultivar; CWIN, cell wall invertase; CIN, cytoplasmic invertase; DAF, days after flowering; DHAP, dihydroxyacetone phosphate; DP, degree of polymerization; FBP, fructose-1,6-bisphosphate; FBPase, fructose-1,6-bis-phosphatase; FK, fructokinase; Fru, fructose; F6P, fructose-6-phospate; GAP, glyceraldehyde 3-phosphate; GBSS, granule bound starch synthase; Glc, glucose; G1P, glucose-1-phosphate; G6P, glucose-6-phosphate; HK, hexokinase; HT, heat-treated; INV, invertase; PGI, phosphoglucoisomerase; PGM, phosphoglucomutase; PFK, phosphofructokinase; RERAF, Risoe Environmental Risk Assessment Facility; SBE, starch branching enzyme; SPP, sucrose-phosphate phosphatase; SPS, sucrose-phosphate synthase; SS, starch synthase; Suc, sucrose; Suc-6P, sucrose-6-phposphate; SuS, sucrose synthase; UDP-glc, UDP-glucose; UGPase, UDP glucose pyrophosphorylase; VIN, vacuolar invertase.

The first committed step in starch biosynthesis, however, is the formation of ADP-glucose, which serves as the glucosyl donor for elongating α-1,4-glucosidic chains by starch synthases (SSs) (Ballicora et�al. 2004). Starch is composed of two discrete α-D-glucose homopolymers: amylose and amylopectin. Amylose has a linear structure, as its Glc units are almost exclusively linked by α-1,4 glycosidic bonds. By contrast, amylopectin molecules contain frequent α-1,6 glycosidic linkages. These introduce branch points resulting in clusters of parallel chains appearing in regular intervals along the molecule’s axis (Zeeman et�al. 2010). Together, amylose and amylopectin form insoluble starch granules, of which amylopectin in barley, the model organism investigated in the current study, constitutes about 70%, while the remaining 30% is amylose (Zeeman et�al. 2010). Notably, barley displays a characteristic bimodal starch granule distribution, with larger A-granules (� ca. 25 �m) and smaller B-granules (� ca. 5 �m; Bathgate and Palmer 1973).

Higher plants possess five different classes of SSs—granule bound starch synthase (GBSS), which synthesizes amylose and soluble SSs, named SSI to SSIV, involved in amylopectin biosynthesis (Zeeman et�al. 2010). The α-1-6-linkages are introduced through starch branching enzymes (SBEs) (Tetlow and Emes 2014), of which three isoforms, SBEI, SBEIIa and SBEIIb, are found in the cereal endosperm. Notably, it is technically challenging to access the activity of the individual enzymes in plant extracts because enzymes of the same class, SSs or SBEs, share substrates and each enzyme catalyze the formation of a complex mixture of products. Thus, studies have focused on assessing the combined SS and SBE activity during grain development under physiological (Zheng et�al. 2017) or heat stress (Wallwork et�al. 1998, Ahmed et�al. 2015) conditions. An alternative is a detailed analysis of the starch itself. Recent publications in barley (K�llman et�al. 2015) and wheat (Kalinga et�al. 2014) have shown that analyzing the size of the linear Glc chains obtained after debranching amylopectin offers sufficient resolution to distinguish starch produced at distinct time points during grain filling. Furthermore, several studies succeeded in assigning preferential functions to individual SS and SBE isoforms for different chain subclasses of amylopectin (Jeon et�al. 2010). Therefore, this study utilizes a detailed characterization of chain length distribution of debranched amylopectin in an attempt to infer the state of the starch biosynthetic machinery during the delicate phase of grain filling in plants suffering from a heat wave. These are predicted to increase in occurrence due to climate change (Russo et�al. 2014, Forzieri et�al. 2016).

The current study follows the synthesis of starch in barley directly and in detail and describes heat-induced changes in starch quality and quantity. These findings are complemented with an assessment of the carbohydrate supply to the grain through direct measurements of the relevant sugars and through determining the activity signature of those enzymes involved in mediating Suc influx into the grain. This highlights physiological traits that could be addressed in breeding programs to optimize grain filling under heat stress conditions. Evidently, the major factor contributing to decreased grain starch content observed was precocious senescence induced by the heat wave. The heat stress further directly induced changes in the amylopectin chain length distribution pattern, which finally translated into a higher starch GT. Notably, pronounced changes in amylopectin structure occurred after the termination of starch accumulation independent of the respective growing conditions.

## Results

The current study concerns the effect of a heat wave during grain filling on sink-related metabolic processes, in particular starch biosynthesis and Suc import, in barley (*Hordeum vulgare* L.) grains. Two cohorts of barley plants were grown, one subjected to a heat wave starting at 16 days after flowering (DAF) and lasting for five consecutive days (heat treated, HT), and one constantly grown under control conditions mimicking a summer in temperate Northern European climate (control). Samples were taken throughout the grain filling period and from completely yellowed and desiccated mature plants. As increased ambient temperature can accelerate development, the observations in this study are plotted on the basis of thermal time in degree-days after anthesis [�Cd] instead of DAF as described in Wallwork et�al. (1998).

### A heat wave during grain filling decreases starch content in barley grains

The effect the HT had on grain weight and starch accumulation is shown in Fig.�2. The heat wave caused a decrease in grain weight (Fig.�2A) by approximately 25%. Notably, HT plant grain weight is lower during and just after the treatment, but equal to control plants between 500 and 550�Cd, while it deviates again later during development.

**Fig. 2:**
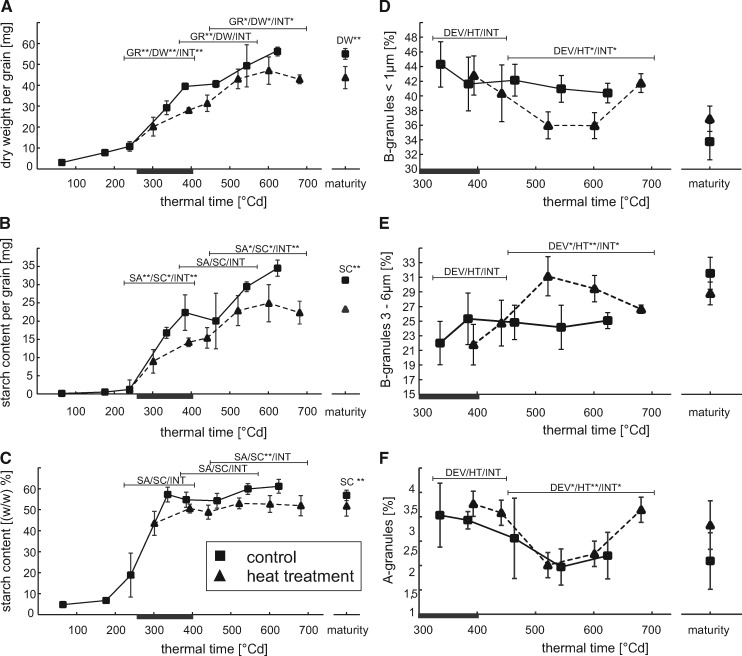
The impact of a heat wave during the grain filling period on starch accumulation and starch granule size distribution in barley cultivar Quench. The duration of the heat wave is indicated by a black bar on the abscissae of each graph. Squared symbols represent data points from plants grown under control conditions, while triangles represent plants that suffered from exposure to a heat wave between days 16 and 21 after anthesis. Time is shown as thermal time (�Cd) to facilitate comparison between the two curves shown. Samples were taken during grain filling as well as from mature plants that underwent complete senescence. (A) Average grain weight in mg. (B) Starch content per grain in mg (C) Relative starch content of the grain [(w/w)%] (D) Proportion of B-granules <1�m (E) Proportion of B-granules 3–6�m (F) Proportion of A-granules. For samples taken during development (*n* = 3), for samples taken at maturity (*n* = 5). Starch granule measurements were made in 3 technical replicates per sample. The bars above the respective curves indicate ANCOVA analysis showing *P < 0.05 or **P < 0.01 significance. The symbols * and ** indicate the same significance thresholds for ANOVA analysis of the mature samples. GR, growth rate representing difference in grain weight over time of combined treatments; DW, dry weight representing differences in grain weight between treatments; SA, starch accumulation representing differences in starch content in thermal time of combined treatments; SC, starch content representing differences in starch content between treatments; DEV, difference in starch granule content over time of combined treatments; HT, difference in starch granule content between treatments; INT, interaction between changes over the indicated thermal time period and the applied treatment.

Starch accumulation commences after 176�Cd and accelerates after 240�Cd (Fig.�2B, C). Grain starch content is consistently lower in HT samples when referring to thermal time. However, when DAFs are considered, the starch content in HT plants is barely different from that of control plants after the heat wave and until after starch accumulation stalls in HT plants (Supplementary Fig. S1A). A similar observation has been made by Wallwork et�al. (1998). It indicates that starch accumulation rate in HT plants at this time point is not decreased.

As starch is the largest fraction of cereal grain weight (Thitisaksakul et�al. 2012), starch weight per grain (Fig.�2B) follows total grain weight closely (Fig.�2A). Notably, while starch weight per grain in control samples increases until 624�Cd, starch accumulation ceases 10 d earlier, at 521�Cd, in HT plants.

Amylose content in HT and control samples does not vary in mature samples, but is increased in HT grains at the end of heat treatment as well as after 521�Cd, just before the onset of senescence (Supplementary Fig. S1B). However, GBSSI is neither more abundant during the heat wave nor at the end of the grain filling period in HT samples (Supplementary Fig. S2). In the absence of direct activity measurements, we can only conclude that the differences, if any, are not dramatic.

As plants suffering from the heat wave yellowed and matured early, the chlorophyll content of the flag leaves was determined (Supplementary Fig. S1C). After 521�Cd, almost no chlorophyll was detected in leaves from HT plants, while chlorophyll levels in control plants remained well above detection limit. These measurements confirm early senescence in HT plants, which coincides with the stalling of grain size increase and starch accumulation.

### The heat wave itself does not change the bimodal starch granule size distribution

While A-granules are formed between DAFs 5–10 in barley, B-granules are initiated about 10 d later (Burton et�al. 2002), which coincides with HT in the current study. To understand if HT had an effect on the bimodal distribution of starch granules, A- and B-granules were counted starting from DAF21, which depicts the last sampling during HT, until maturity using flow cytometry (Fig.�2D–F; Supplementary Fig. S3). During and directly after HT, no significant differences in A- and B-granule count could be detected. However, at 521 and 601�Cd a shift in the proportion of B-granules was observed—the fraction of smaller B-granules (<1 �m) was decreased in HT samples (Fig.�2D) while the fraction of larger B-granules (3–6 �m) was increased (Fig.�2E). The content of A-granules remained unchanged during development (Fig.�2F) and in mature samples no differences in A-granule percentage could be observed.

### The biosynthesis of amylopectin is affected by heat stress

The heat wave significantly increased the GT in starch obtained from mature grains while the melting enthalpy was reduced (Table�1), indicating heat-induced structural changes within the starch are not due to varying proportions of amylose. To investigate starch structure in detail, the chain length distribution of debranched amylopectin was analyzed (Supplementary Table S1). In the following, degree of polymerization (DP) refers to the number of linearly linked α-1,4-linked Glc units in each chain after debranching amylopectin at its α-1,6-linkages.

**Table 1:** Peak GT, melting enthalpies and amylose contents of starch from mature barley grains grown under control and heat treatment conditions determined by differential scanning calorimetry

	Average peak GT (*n* = 5) (�C)	Standard deviation	Melting enthalpy (J/g)	Standard deviation	Amylose content (% of starch)	Standard deviation
Control	62.0^a^	0.2	4.8^c^	0.5	22.8	1.2
Heat treatment	64.6^b^	0.1	3.4^d^	1.1	22.5	1.0

^a/b^ Different letters indicate that values differ significantly (*P* < 0.0001).

^c/d^ Different letters indicate that values differ significantly (*P* < 0.05).

Amylopectin from mature HT samples showed a decrease in the abundance of DP 7–11 and an increase of DP 20–37 (Fig.�3E), which is known to correlate with an increase in GT (Nakamura et�al. 2002, Vamadevan et�al. 2013), as well as an increase in DP 12–15. A schematic representation of those changes is presented in Supplementary Fig. S4. Direct comparisons between corresponding samples with the most similar thermal times (Fig.�3A–D) further show reduced abundance of short chains already at the end of heat treatment and beyond that in HT plants. Notably, differences in amylopectin structure are maximal 3 d after the termination of the heat wave at approximately 450�Cd, with reduced DP 9–13 chains and increased amount of chains larger than DP 14.

**Fig. 3:**
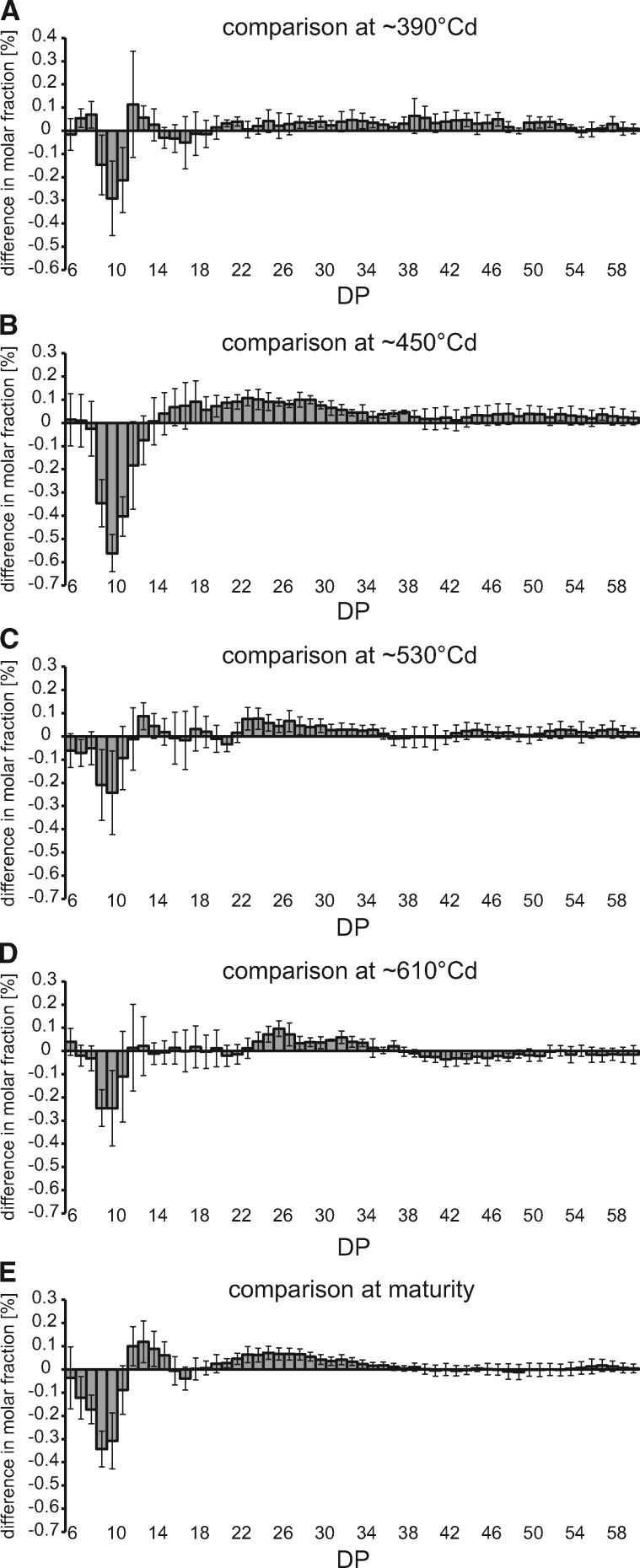
Heat-induced changes in amylopectin structure. Comparison of chain length distributions of debranched amylopectin between grains from plants grown under control and heat wave conditions at time points comparable in terms of �Cd. Positive bars reflect an excess of those chains in heat wave grains. (A) Heat wave at 393�Cd minus control at 384�Cd; (B) heat wave at 441�Cd minus control at 464�Cd; (C) heat wave at 521�Cd minus control at 544�Cd; (D) heat wave at 601�Cd minus control at 624�Cd; (E) heat wave at maturity minus control at maturity.

Changes in chain length distribution are particularly visible when the dynamics of specific DP ranges are followed throughout grain filling. To this end, DP ranges comprising DP 8–11, DP 13–24 and DP 31–60 were chosen (Fig.�4), as SSI, SSIIa and SSIII are predominantly involved in the synthesis of chains of the respective lengths (Morell et�al. 2003, Fujita et�al. 2006, Jeon et�al. 2010). Similarly, decrease in SBEI, HvSBEIIa and HvSBEIIb activities cause deficits in DP 12–22, DP 10–12 and DP 12–18, respectively (Satoh et�al. 2003, Regina et�al. 2006, Regina et�al. 2010, Sun et�al. 2017), and were thus included (Supplementary Fig. S5). However, a clear separation of the individual enzyme activities cannot be achieved by analyzing specific DP ranges, as starch biosynthetic enzymes work in concert to produce starch. Still, these ranges allow comparison of our results to the published data. A statistical assessment of the dynamics in amylopectin structure is shown in Supplementary Table S2.

**Fig. 4:**
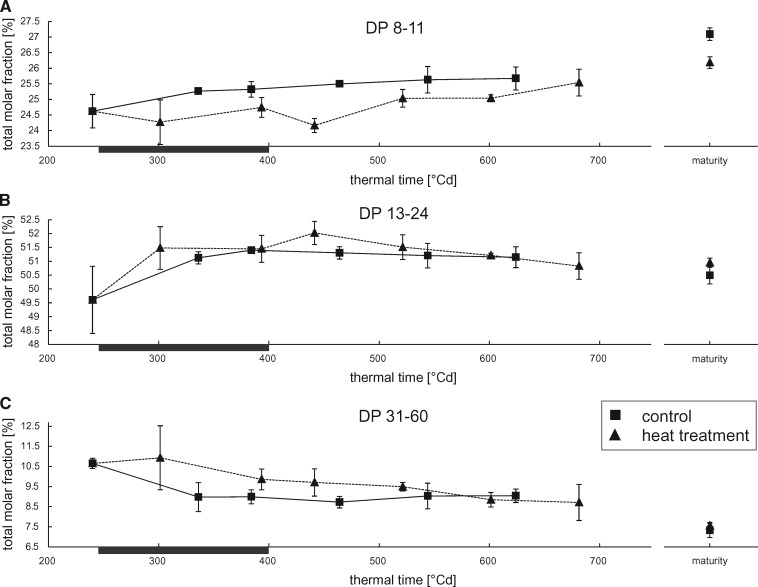
Dynamics of the amylopectin chain length distribution shown as the cumulative molar fractions of DP ranges DP 8–11, DP 13–24 and DP 31–60 during grain filling in barley grown under control and heat wave conditions. Molar fractions depicted are the sum of the molar fractions of individual DPs within the DP range of DP 8–11 (A), DP 13–24 (B) and DP 31–60 (C) after normalization. The duration of the heat wave is indicated by a black bar on the abscissae of each graph. Squared symbols represent data points from plants grown under control conditions, while triangles represent plants that suffered from a heat wave between days 16 and 21 after anthesis. Error bars represent � the standard deviation.

Although representing a small molar fraction, chains in the range of DP 31–60 contribute to a large extent to the mass of amylopectin due to the high number of Glcs per chain. These chains are slightly increased during and after the period of thermal stress before starch accumulation stalls in HT plants.

Chains in the range of DP 13–24 increase in proportion after 240�Cd in both treatments, corresponding to an increase of SSIIa abundance in Western blots after that time point (Supplementary Fig. S2). During the further course of grain filling, the content of chains of DP 13–24, however, does not deviate considerably from each other between treatments, while Western blot analyses revealed a lower abundance of SSIIa directly after the heat treatment and at the end of the grain filling period (Supplementary Fig. S2).

Fig.�4 shows that HT plants had reduced DP 8–11 content compared with control plants. This reduction is significant in mature samples and at 301 and 441�Cd compared to the corresponding controls, when the DP 8–11 content is lowest. At 441�Cd the overlapping HvSBEIIa range of DP 10–12 shows a significant decrease in abundance in samples from HT plants as well (Supplementary Fig. S5). Western blots show SSI to be decreased early during the heat wave at 301�Cd, but not at 393�Cd, and again after the heat wave at 441 and 521�Cd (Supplementary Fig. S2).

Notably, chains of DP 8–11 significantly increase in abundance during transition to maturity (*P* < 0.05) both in control and HT plants (Fig.�4), but they are significantly increased already at 681�Cd in HT plants, probably due to the earlier onset of senescence. Fig.�5 shows in detail how the earlier termination of grain filling influences the dynamics of amylopectin chain length distribution. In control plants, the amylopectin structure remains largely the same between 384 and 624�Cd (Fig.�5A–D), even though the total starch content per grain is more than double during that period. However, while no additional starch is deposited in the grains between 624�Cd and maturity (Fig.�2), the starch structure changes. Chains of DP 2–14 increase in abundance by a cumulative 3.57% with a maximum of 0.52% for DP 10, while longer chains decrease in abundance correspondingly. In HT plants, however, the changes within amylopectin between the last sampling time point (681�Cd) and maturity are less pronounced (Fig.�5E). Notably, the comparison of DP profiles in mature HT samples with samples harvested at 521�Cd (Fig.�5G and insert) is almost identical to that of control plants between maturity and 624�Cd, both in magnitude and DP profile. In HT plants, 521�Cd is the time point when starch accumulation stalls (Fig.�2).

**Fig. 5:**
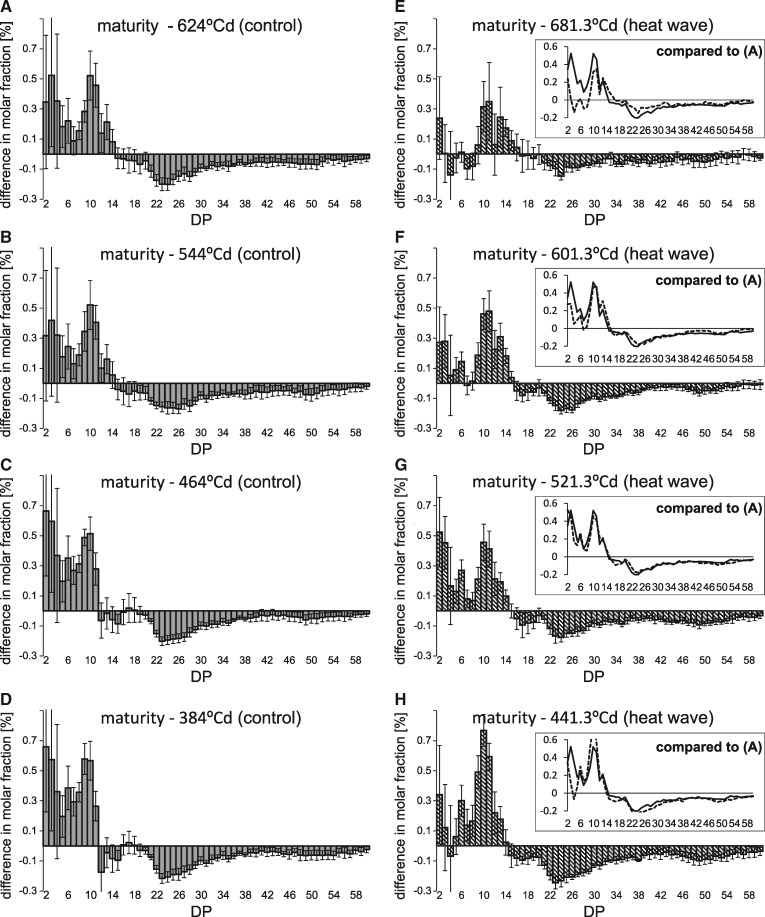
Changes in amylopectin structure during grain filling in comparison to maturity. Comparison of chain length distributions of debranched amylopectin between mature grains and developing grains from plants grown under control and heat wave conditions. Positive bars reflect an excess of those chains in mature grains. (A–D) The comparison between samples taken from mature plants and at 624, 544, 464 and 384�Cd for control conditions (filled bars). (E–H) The comparison between mature samples and those taken at 681, 601, 521 and 441�Cd from heat HT (striped bars). Inserts in (E–H) show a direct comparison between the respective chain length distribution to that in (A), where the solid line is the profile as in (A) and the dotted line is the profile as in the respective panel.

### The heat wave only had minor effects on the composition of the cell wall

A comprehensive microarray polymer profiling (CoMPP) was used to detect specific cell wall components (Supplementary Fig. S6). Mainly β-glucan, mannan and arabinoxylan could be detected. A relatively increased arabinoxylan content was observed in mature HT grains, while neither β-glucan nor mannan levels were affected. During development, however, the β-glucan content increased relatively faster in the HT plants between 400 and 650�Cd while mannans were decreased from 400 to 650�Cd (Supplementary Fig. S6).

### Effect of a heat wave on Suc levels in developing barley grains

Ultimately, all starch and cell wall components produced in the grain originating from photo-assimilates is transported there as Suc. To understand how Suc supply is affected by HT, Suc content was quantified starting at 240�Cd (Fig.�6). It decreases between 240 and 464�Cd in control plants by almost 70% and then remains at constant levels (Fig.�6). By contrast, Suc concentrations in HT plants decreased directly after the onset of HT and remain decreased after 5 d of thermal stress (393�Cd). After that, however, Suc content is elevated in grain samples from HT plants.

**Fig. 6:**
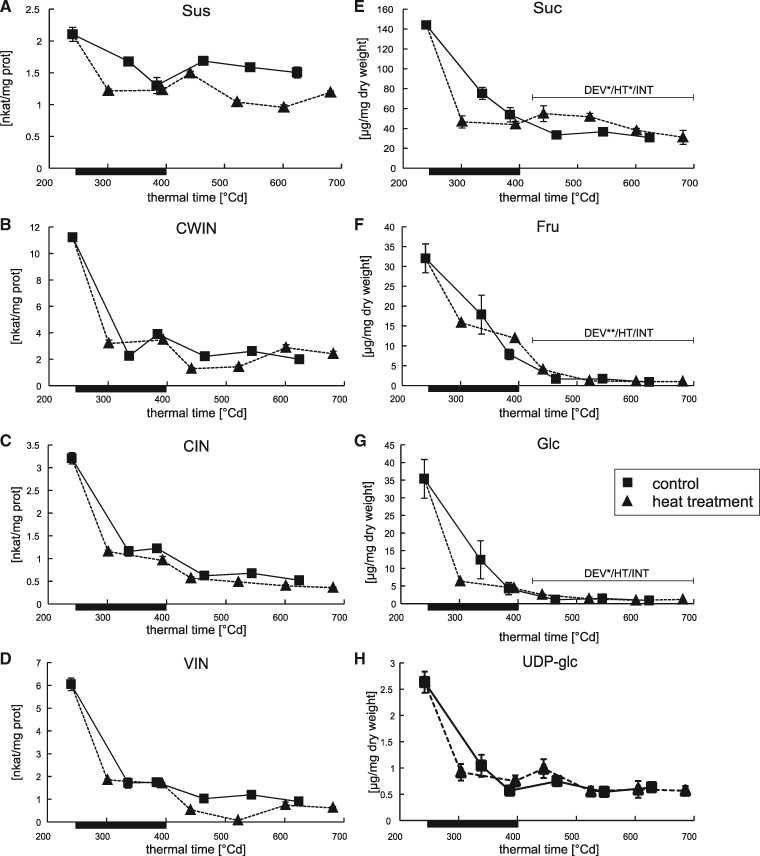
Time course analyses of the activity of sucrolytic enzymes and the content of their substrate and reaction products in the barley grain. Depicted is the phase of starch accumulation (starting with DAF 15, 240�Cd). The duration of the heat wave is indicated by a black bar on the abscissae of each graph. Squared symbols represent data points from plants grown under control conditions, while triangles represent plants that suffered from a heat wave between days 16 and 21 after anthesis. Time is shown as thermal time [�Cd] to facilitate comparison between the two curves shown. Enzyme activities are shown in nkat per mg protein on the left hand side for (A) SuS, (B) CWIN, (C) CIN and (D) VIN. For analysis, material from three biological replicates was pooled. Error bars represent � the standard deviation of three measurements of the combined sample. On the right hand side, the grain content of (E) Suc, (F) Fru, (G) Glc and (H) UDP-glc are shown in micrograms per milligram dry weight. The same samples as in (A–D) were used for analyses, but measurements were done on individual samples. Error bars represent � standard deviations between biological replicates, *n* = 3. The bars above the respective curves indicate ANCOVA analysis showing *P < 0.05 or **P < 0.01 significance. DEV, difference in sugar content over time of combined treatments; HT, difference in sugar content between treatments; INT, interaction between changes over the indicated thermal time period and the applied treatment.

Notably, the relative accumulation of Suc in grains of HT plants after 393�Cd parallels with reduced activities of the main sucrolytic enzymes SuS, and all three INV isoenzymes CWIN, CIN and VIN (Fig.�6A–D). This observation was confirmed by additional experiments (Supplementary Fig. S7), as the original activity signatures were obtained from pooled samples. The HT induced repression of INV activity preceded their developmentally regulated repression. Still, while SuS activity in HT plants appeared to remain decreased compared with control plants, CWIN and VIN resumed the activity levels of the control plants.

Interestingly, activity levels of ADP-glucose pyrophosphorylase (AGPase), the first committed step of starch biosynthesis dropped only initially during HT, but this was followed by stable levels towards the end of the HT and a recovery immediately after, thus that activities in grains from control and HT plants were similar after 400�Cd (Supplementary Table S3).

### Effect of a heat wave on carbon metabolism of barley flag leaf

Physiological and metabolic profiling of grains was complemented with the analysis of flag leaves from the same plants (Supplementary Table S3) as flag leaves are the main source of Suc transported to sink tissues in cereals (Gordon et�al. 1980). Flag leaf Suc content was found to be reduced in HT plants from the onset of the heat wave and remained constantly below control levels throughout the course of the experiment (Fig.�7D) contrary to the findings in grains where levels were decreased during the heat wave but elevated directly thereafter. Further, Glc and Fru levels were monitored (Fig.�7E, F). After a temporary depletion during HT (240–393�Cd), hexose levels increased compared with control plants immediately afterwards and reached control levels by 521�Cd.

**Fig. 7:**
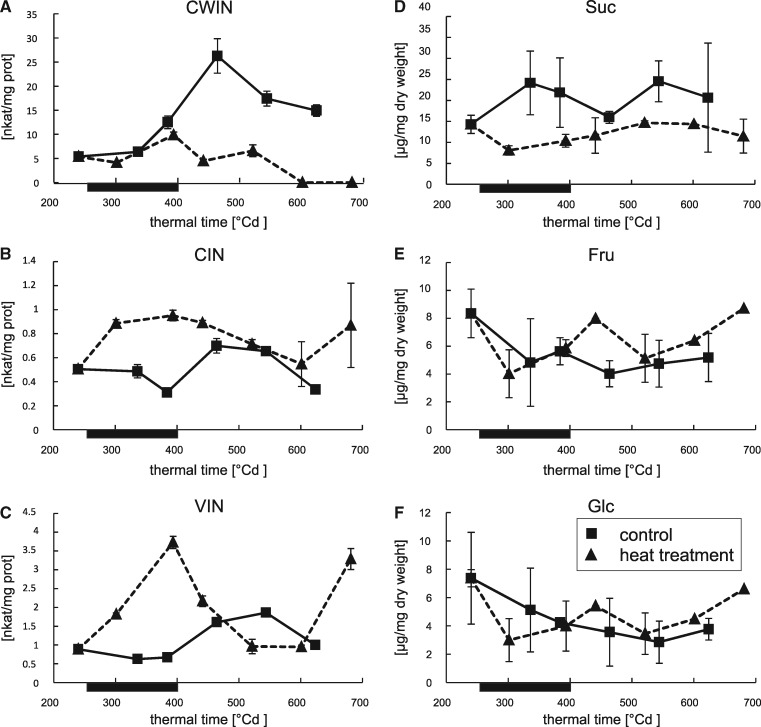
Effect of the heat wave on the sucrolytic enzymes and the content of their substrate and reaction products in the in flag leaves. Depicted is the phase of starch accumulation (starting with DAF 15, 240�Cd). The duration of the heat wave is indicated by a black bar on the abscissae of each graph. Squared symbols represent data points from plants grown under control conditions, while triangles represent plants that suffered from a heat wave between days 16 and 21 after anthesis. Time is shown as thermal time (�Cd) to facilitate comparison between the two curves shown. Enzyme activities are shown in nkat per mg protein for (A) CWIN, (B) CIN, (C) VIN and (D) Suc, (E) Fru, (F) Glc. For analysis, material from three biological replicates was pooled. Error bars represent � the standard deviation of three measurements of the combined sample.

Parallel to the analysis in grains, the activity signatures of the different INVs were determined in flag leaves as well. Both CIN and VIN showed a very strong, transient increase in activity during the heat wave between 240 and 393�Cd (Fig.�7B, C). The activity of CWIN in HT plants, on the contrary, is not changed during HT when compared with control. However, later during grain development, between 384 and 464�Cd, CWIN activity more than doubled in control plants and remained elevated in comparison to earlier time points, while no such increase was observed in HT plants, where it declined to non-detectable levels after 521�Cd.

The measurements of INV activity were part of an analysis that determined activity signatures for 13 key enzymes involved in carbohydrate metabolism. The complete analysis is shown in Supplementary Table S3.

## Discussion

The current study suggests to separate grain development in response to a heat wave into three distinct stages: (i) the heat wave itself, when grain weight increases more slowly and starch accumulation decelerates, (ii) a period of increase in grain weight following the return to a normal temperature regime and (iii) the process of maturation and desiccation.

The first stage, during the heat wave itself, is characterized by HT plants gaining less grain weight and starch than control plants. Notably, the proportion of A- and B-granules does not change during this period arguing against an effect of the heat wave on B-granule initiation happening at this stage (Burton et�al. 2002). Possibly, heat sensitivity of starch biosynthetic enzymes contributes to the observed decrease in starch accumulation. During the heat wave, a reduction in amylopectin chains of DP 8–11 was observed, which is compatible with a reduction in the activities of SSI and SBEIIa alone or combined; although in the absence of direct measurements of the activities of the individual isozymes, any such conclusions must remain speculative. Wallwork et�al. (1998) have shown that the combined soluble SS activity in barley is reduced during and directly after HT, while the combined starch branching activity is decreased directly after HT. Reductions in the relative activity of HvSSI (Cuesta-Seijo et�al. 2016) and of SBEIIa and SBEIIb from rice and maize (Ohdan et�al. 2010) at elevated temperatures have been documented in vitro. Proteomics studies in wheat have further shown a decrease in the abundance of SBE enzymes in HT plants (Zhang et�al. 2017). The SSs from barley (Cuesta-Seijo et�al. 2016) and the BEs from rice and maize (Ohdan et�al. 2010) have been shown to retain activity in vitro in spite of incubation at 40 or 35�C respectively, although those incubations lasted for minutes, not days. Thus, we cannot discard the possibility of protein denaturation in the present study, which could not be ascertained using Western blots. Still, even though the structure of amylopectin is changed during the heat wave, larger changes occurred in the second stage after the heat wave had terminated, around 450�Cd. Thus, the effect of heat wave on starch is extended in time beyond the period of increased temperature. These long lasting changes would be consistent with changes in the abundance of SSs as shown in Supplementary Fig. S2. Yet, a conclusive proof of the causes would require direct measurement of the individual protein activities and their substrate selectivity at the temperatures of the control and treated plants.

Further, the reduced grain weight increase is paralleled by decreased activities of sucrolytic enzymes (INVs and SuS) as well as Suc, Glc and Fru levels in the grains. These obviously reduced Suc supplies to the grain are hence a very plausible explanation for reduced starch content during HT.

Notably, the stage between the termination of the thermal stress and stalling of starch accumulation, is in contrast to the heat wave, characterized by increased Suc levels in HT grains. A comparable observation was made by Wallwork et�al. (1998), which the authors interpreted as decreased Suc consumption in developing grains, a theory reinforced by a parallel strong reduction in AGPase activity. The authors therefore concluded that starch accumulation is sink limited during this period. The decreased activities of the sucrolytic enzymes, CWIN, CIN and VIN, observed in the current study are consistent with this assumption. Sucrolytic activity is a prerequisite for sink efficiency and especially CWIN has been identified as a major determining factor of sink strength. During grain filling, CWIN activity is crucial for establishing high grain weight, as shown for the CWINs GIF1 in rice (Wang et�al. 2008) and Miniature1 in maize (Chourey et�al. 2012, Li et�al. 2013).

However, despite the apparent tailback of Suc after termination of the heat wave, starch accumulation is accelerated again during this second stage in HT plants, which argues against a decrease in sink efficiency. Further, in contrast to the findings of Wallwork et�al. (1998), no meaningful reduction in AGPase activity was observed after termination of the heat wave, which is consistent with a normal starch synthesis capacity in grains at the time. Also, studies in rice found only a moderate reduction in AGPase gene expression under heat stress (Yamakawa et�al. 2007, Tanamachi et�al. 2016), while other studies looking at the activity itself found no reduction, just an offset in time, in heat stressed rice (Ahmed et�al. 2015) and a moderate reduction in wheat (Bansal et�al. 2013).

An alternative explanation for the accumulation of Suc in grains could be increased mobilization of Suc from source tissue. Notably, in flag leaves of HT plants, CWIN activity remained unaltered after HT while in control plants it increased by >100% at the respective developmental stage. The increase of CWIN activity in mature leaves has been described before (Kingston-Smith et�al. 1999) and appears to be both sufficient and necessary for delaying leaf senescence (Balibrea Lara et�al. 2004, Jin et�al. 2009).

Still, an increase of CWIN activity in mature leaves appears counterintuitive given the anabolic function of photosynthetic tissue to produce and export Suc (Schnyder 1993). Zwack and Rashotte (2013) suggested that increased CWIN activity could provide a less productive leaf with an artificially strong source identity. Elevated CWIN activity induces a futile cycle of Suc export to the apoplast, followed by its hydrolysis and the reimport of hexoses. Accordingly, the absence of increased CWIN activity in flag leaves could result in extra supply of Suc to the grain.

Regarding the fine structure of amylopectin, the differences between HT and control plants are maximal at approximately 450�Cd, despite unchanged starch granule proportions at this time point. In particular, decreases in the relative frequency of DP <12 and increases in DP 13–24, observed at approximately 450�Cd, as well as in mature grains, are expected to result in increased GT (Fujita 2015). The fact that changes are maximized days after the HT can be explained either by regulatory effects on either the abundance or activity of starch biosynthetic enzymes or by the continued effects of an altered amylopectin structure, which provides a different template for further elongation of amylopectin.

The third stage in grain development in response to the HT, corresponding to the grain desiccation process, is characterized by a cessation of grain weight increase. In fact, a slight decrease in dry grain mass was found. The phase of desiccation was captured in a single data point in control plants, but spread over three time points for HT plants. This was also illustrated by a change in starch granule proportions. HT samples show a significantly decreased content of very small B-granule as well as an increased content of B-granules of 3–6 �m, which can be interpreted as a lack of B-granule initiation in HT samples due to the precocious senescence.

Notably and unexpectedly, the most profound changes in amylopectin chain length distribution were observed in this period. In control plants, a relatively large increase of short chains in the range of DP 2–14 and a decrease of chains larger than DP 14 occurred between 624�Cd and harvest maturity i.e. after grain growth had ceased. In HT plants, very similar structural changes occurred between 521�Cd and maturity i.e. after starch accumulation had terminated. This suggests that the processes involved are identical but just displaced forward in time for HT plants. Finding such large changes in a period where growth had ceased was unexpected. Previous studies focused on the period up to cessation of grain growth in wheat (Kalinga et�al. 2014) or prior to it in barley (K�llman et�al. 2015) and hence did not provide data on starch modification during desiccation. As the amount of starch per grain slightly declined between 521�Cd (or 624�Cd, respectively) and maturity, it is possible that amylolytic or debranching enzymes induced structural change. Another plausible cause for the changes observed between the cessation of grain growth and harvest maturity are the branching enzymes. During this period, no new sugars are supplied to the grains and hence no Glc units are added to the starch molecule. However, branching enzymes can retain activity as their substrate, amylopectin, remains available (Tetlow and Emes 2014). SBE activity always results in the disruption of a long chain of Glcs to produce two shorter ones (Nakamura et�al. 2010), thus leading to a reduction in the amount of longer chains and an increase in the frequency of shorter chains, which is observed in this study. This effect is also present during starch growth but it is masked by the elongation activities of SSs. A schematic representation of the competing effects is presented in Supplementary Fig. S4. In particular, SBEI activity might be causative for most of the changes to amylopectin during maturation. SBEI has a relatively late expression peak in barley (Radchuk et�al. 2009), thus its relative abundance and relevance could be maximized during the desiccation process. Rice SBEI preferentially catalyzes the formation of DP 10–12 chains when supplied with amylose or modified amylopectin as a substrate in in vitro experiments (Nakamura et�al. 2010). In our current study, these chains increased most in abundance during desiccation. SBEIIa has been observed to have a similar effect in barley (Regina et�al. 2010), but that effect was while including the simultaneous action of SSs. In rice, SBEIIa and SBEIIb preferentially produce chains of DP 6–7 in the absence of elongation activities (Nakamura et�al. 2010). These chains are less abundant, thus arguing against a dominant role of these enzymes during desiccation. Overall, the starch in mature grains from HT plants is characterized by a decrease in the relative frequency of DP < 12 and an increase in DP 13–24 when compared with control plants, which is expected to result in increased GT (Fujita 2015). The observed differences between HT and control plants are potentially a carryover effect from early grain development, as similar changes were observed at approximately 450�Cd.

In summary, the comparable responses in grain weight, starch content and starch structure during the desiccation phase suggest that the processes are very similar in HT and control plants. Thus, this stage does not add to yield or quality losses other than through its 10 d early onset in stressed plants. Consequently, the most promising strategy to improve the performance of cereal crop plants under thermal stress appears to be source related, namely the introduction of functional stay-green traits that prevent precocious senescence. In sorghum, stay green has for instance been associated with the formation and presence of the cyanogenic glucoside dhurrin (Hayes et�al. 2015, Emendack et�al. 2017). Barley leaves also contain hydroxy nitrile glucosides including the cyanogenic glucoside epiheteroendrin (Knoch et�al. 2016).

## Material and Methods

### Plant material and experimental growth conditions

*Hordeum vulgare* L. cultivar Quench was grown (density: 8 plants/11 L pot) at the RERAF (Risoe Environmental Risk Assessment Facility) phytotron in Roskilde, Denmark (Frenck et�al. 2011, Ingvordsen et�al. 2015) under 16 h/8 h long-day conditions with temperature and relative humidity set to 18�C/70% (day) and 12�C/55% (night). Heat treatment (HT) was applied from 16 to 21 DAF, at 33�C (day) and 26�C (night), while the other parameters were kept constant. Water supply was not limited during HT and water was given until the spike on the first developing tiller had fully yellowed. Spikes were left on the culm to desiccate before harvest then further dried at 25�C for 3 d.

### Sampling of plant material

Pre-experiments established a correlation between the visible length of awns emerging from the flag leaf sheath and the DAF. The DAF of the first developing tiller was determined for every plant and the mean was used as ‘day 0’ for all plants (Wallwork et�al. 1998). Sampling of complete ears and flag leaves of the first tiller was done at DAFs 4, 11, 15, 18, 21, 24, 29, 34 and 39. HT DAF 21 samples were collected under HT conditions. Samples were flash frozen in liquid nitrogen and stored at −80�C. Material from DAF 18 control plants was lost. Further samples were taken from fully matured and dried plants and kept at room temperature.

Conversion of DAF into degree days [�Cd] was done according to Equation (1), where *L* represents the number of days, TL the average temperature during the day and TB the base temperature, the temperature below which plant development stops. TB for barley grown in temperate regions can be considered 0�C.

(1) �Cd=∑L(TL-TB)

### Measurement of starch GT and melting enthalpy

GT was determined as peak temperature by differential scanning calorimetry on a Mettler Toledo DSC1 instrument (Mettler Toledo, Greifensee, Switzerland) in three technical replicates on five biological replicates per treatment. Melting enthalpies was determined with the same software. A two-sided *t*-test for two independent populations with equal variance assumption was done using Microsoft Excel.

### Determination of grain starch content and quantification of amylose and β-glucan content

For frozen samples, all filled grains from one side of the rachis were pooled while kept frozen. Grains were lyophilized at −15�C for 5 d followed by 24 h at room temperature. All grains from matured, dried samples were lyophilized at room temperature (24 h). Dried grains were crushed, cooled in liquid nitrogen and ground to flour in a SPEX SamplePrep2010 Geno/Grinder� (Metuchen, New Jersey, USA).

Determination of total starch was done using the AA/AMG kit from Megazyme (Bray, Ireland). D-Glucose (1 mg/ml) and Regular Maize Starch from Megazyme (Bray, Ireland) served as controls. Amylose content and amylose: amylopectin ratio was determined using the K-AMYL kit (Megazyme, Ireland). β-glucans were quantified using the K-BGLU kit (Megazyme, Ireland). Five milligram aliquots of flour were used, otherwise manufacturer’s recommendations were followed. Grubbs test for outliers, ANOVA and ANCOVA were made using XLSTAT add-on for Microsoft Excel (Addinsoft, New York, NY, USA). ANOVA was applied to mature samples and ANCOVA to distinct stages during development (HT: 240–393�Cd; recovery: 384–601�Cd; desiccation: 464–681�Cd). For samples taken during development *n* = 3, for samples taken at maturity *n* = 5 plants were used for measurements.

### Determination of relative A-granule content in barley starch

Starch granules size distribution was measured using flow cytometry on a NovoCyte� instrument (ACEA Biosciences, Inc., San Diego, CA, USA) set to λ = 480nm (Forward scatter threshold: 6,000). Per sample 50,000 events were counted similar as in Bull et�al. (2018) in three technical replicates. ANOVA (for mature samples) and ANCOVA (direct HT effect: 336–441�Cd; period until desiccation: 464–681�Cd) were made using XLSTAT add-on for Microsoft Excel (Addinsoft). Three biological replicates were used for samples taken during development and five for mature samples.

### Chlorophyll determination

Flag leaves were ground using a SPEX SamplePrep2010 Geno/Grinder� (Metuchen, New Jersey, USA). Powder was dissolved in methanol, centrifuged and supernatants were analyzed at 665.2 and 652.4 nm using a Biochrom WPA Biowave II UV/Visible Spectrophotometer (Cambourne, UK). Results were normalized to fresh weight (*n* = 3 for samples taken during development; *n* = 5 for mature samples).

### Analysis of chain length distributions in debranched amylopectin

Chain length distribution of debranched amylopectin was determined from the same samples used to determine grain starch, amylose and β-glucan content. Starch was isolated from flour (2 mg) following Shaik et�al. (2014). Starch was debranched with *Pseudomonas spearoides* isoamylase and *Bacillus licheniformis* pullulanase (Megazyme, Ireland) and analyzed in an ICS-3000 chromatography system (Dionex) using CarboPac PA100 analytical columns following Blennow et�al. (1998). Peak areas were converted to molar ratios based on comparison with a debranched oyster glycogen standard (type II, Sigma, Denmark). All molar fractions reported are after normalization to 100% based on the accumulated molar amounts from DP (degree of polymerization) 6 to DP 35. Standard deviations for the differences between molar fractions were calculated as the square root of the sum of the squares of the standard deviations of each group of samples, weighted when the number of samples was uneven between groups. ANOVA including Tukey's range test on specific DP ranges in amylopectin molecules (Supplementary Table S2) was performed using XLSTAT add-on for Microsoft Excel (Addinsoft). (Biological replicates: *n* = 3 for samples taken during development; *n* = 5 for mature samples.)

### Comprehensive microarray polymer profiling

Samples (10 mg) were de-starched with Termamyl� (Novozymes, Denmark) and washed twice with 70% (v/v) ethanol and with acetone. Samples were extracted as described in Moller et�al. (2007) and processed as described in Pedersen et�al. (2012) in four technical replicates and four dilutions, biological replicates were *n* = 3 for samples taken during development; *n* = 5 for mature samples.

### Enzymatic assays

Pooled grains from one side of the rachis and the corresponding flag leaf of the same plant were ground to powder (*n* = 3). Aliquots of biological replicates (100 mg) were pooled prior to enzymatic assays, which were performed as described in Jammer et�al. (2015). For selected enzymes, additional measurements on individual plants (*n* = 3) were added. ANCOVA tests were made using XLSTAT add-on for Microsoft Excel (Addinsoft) on distinct stages during development (HT: 240–393�Cd; recovery: 384–544�Cd; desiccation: 464–681�Cd). Different plant individuals were used for enzymatic analysis and the characterization of starch.

### Metabolite extraction

Metabolite extraction was performed on the same material (ca. 100 mg) as used for the enzymatic assays following Lunn et�al. (2006) (*n* = 3) with few modifications. Specifically viscous, high molecular-mass components were removed from the samples with SEP-Pack Cartridges (Waters, Milford, Massachusetts, USA) and a 0.2 μm Supor� membrane filter (PALL, New York, NY, USA). Lactose was added to the frozen tissue slurry immediately after the addition of cold CHCl_3_/CH_3_OH.

### Assay of metabolites

Aliquots (10 μl) of grain extracts (*n* = 3) were analyzed in an ICS-3000 Dionex chromatography system as for the chain length distributions. For Suc, Glc and Fru, the mobile phases were (A) water and (B) 0.5 M NaOH. Compounds were eluted with a multi-step gradient: 100% A (0–10 min), 30% B (10–20 min), 30–70% B (20–25 min), 70–100% B (25–31 min). For the analysis of UDP-glc the solutions used were: (A) 75 mM NaOH and (B) 75 mM NaOH + 500 mM NaOAc. Multi-step gradient was as follows: 100% A (0–10 min), 100–70% A (10–20 min), 100–62% A (20–40 min), 62–59% A (40–70 min), 100% B (70–75 min), 0–100% A (75–77 min), 100% A (77–85 min). Peak areas were integrated with Chromeleon 6.8 (Dionex), and processed in Microsoft Excel. Quantification of Suc, Fru and Glc from leaf samples was performed similarly with CarboPac SA10-4 �m analytical columns in a Dionex ICS-5000+ Reagent-Free HPLC System (Dionex, Thermo Fisher Scientific, Waltham, MA, USA) with PAD detection. Monosaccharides were separated with a 5 mM KOH isocratic condition for 30 min, quantified by comparison with authentic standards and normalized to dry weight.

## Funding

The Carlsberg Foundation [2014_01_0013, CF15-0476, CF16-0506]; the European Union’s Horizon 2020 research and innovation program under the Marie Skłodowska-Curie action [grant agreement No 722338 (PlantHUB)]; the Innovation Fund Denmark [5112-00006B]; a VILLUM Foundation grant [VKR023054/00007523] to the VILLUM Center for Plant Plasticity and a grant from the University of Copenhagen UCPH Excellence Program for Interdisciplinary Research to the Center for Synthetic Biology ‘bioSYNergy’.

## Supplementary Material

pcz155_Supplementary_Figures-Tables

## References

[pcz155-B1] Ahmed N., Tetlow I.J., Nawaz S., Iqbal A., Mubin M., Nawaz Ul Rehman M.S. (2015) Effect of high temperature on grain filling period, yield, amylose content and activity of starch biosynthesis enzymes in endosperm of basmati rice. J. Sci. Food Agric. 95: 2237–2243. 25284759 10.1002/jsfa.6941

[pcz155-B2] Asaoka M., Okuno K., Fuwa H. (1985) Effect of environmental temperature at the milky stage on amylose content and fine structure of amylopectin of waxy and nonwaxy endosperm starches of rice (*Oryza sativa* L.). Agric. Biol. Chem. 49: 373–379.

[pcz155-B3] Balibrea Lara M.E., Gonzalez Garcia M.-C., Fatima T., Ehne� R., Lee T.K., Proels R., et al (2004) Extracellular invertase is an essential component of cytokinin-mediated delay of senescence. Plant Cell 16: 1276–1287. 15100396 10.1105/tpc.018929 PMC423215

[pcz155-B4] Ballicora M.A., Iglesias A.A., Preiss J. (2004) ADP-glucose pyrophosphorylase: a regulatory enzyme for plant starch synthesis. Photosynth. Res. 79: 1–24. 16228397 10.1023/B:PRES.0000011916.67519.58

[pcz155-B5] Bansal K., Munjal R., Madan S., Arora V. (2013) Influence of high temperature stress on starch metabolism in two durum wheat varieties differing in heat tolerance. J. Wheat Res. 4: 43–48.

[pcz155-B6] Bathgate G., Palmer G. (1973) The *in vivo* and *in vitro* degradation of barley and malt starch granules. J Inst. Brew. 79: 402–406.

[pcz155-B7] Bita C.E., Gerats T. (2013) Plant tolerance to high temperature in a changing environment: scientific fundamentals and production of heat stress-tolerant crops. Front. Plant Sci. 4:10.3389/fpls.2013.00273 PMC3728475 23914193

[pcz155-B8] Blennow A., Bay-Smidt A.M., Wischmann B., Olsen C.E., M�ller B.L. (1998) The degree of starch phosphorylation is related to the chain length distribution of the neutral and the phosphorylated chains of amylopectin. Carbohydr. Res. 307: 45–54.

[pcz155-B9] Bull S., Seung D., Chanez C., Mehta D., Kuon J.-E., Truernit E., et al (2018) Accelerated ex situ breeding of GBSS- and PTST1-edited cassava for modified starch. Sci. Adv. 4: eaat6086. 30191180 10.1126/sciadv.aat6086 PMC6124905

[pcz155-B10] Burton R.A., Jenner H., Carrangis L., Fahy B., Fincher G.B., Hylton C., et al (2002) Starch granule initiation and growth are altered in barley mutants that lack isoamylase activity. Plant J. 31: 97–112. 12100486 10.1046/j.1365-313x.2002.01339.x

[pcz155-B11] Chourey P.S., Li Q.-B., Cevallos-Cevallos J. (2012) Pleiotropy and its dissection through a metabolic gene Miniature1 (Mn1) that encodes a cell wall invertase in developing seeds of maize. Plant Sci. 184: 45–53. 22284709 10.1016/j.plantsci.2011.12.011

[pcz155-B12] Chun A., Lee H.-J., Hamaker B.R., Janaswamy S. (2015) Effects of ripening temperature on starch structure and gelatinization, pasting, and cooking properties in rice (*Oryza sativa*). J. Agric. Food Chem. 63: 3085–3093. 25781203 10.1021/jf504870p

[pcz155-B13] Cuesta-Seijo J.A., Nielsen M.M., Ruzanski C., Krucewicz K., Beeren S.R., Rydhal M.G., et al (2016) In vitro biochemical characterization of all barley endosperm starch synthases. Front. Plant Sci. 6: 1265. 10.3389/fpls.2015.01265 PMC4730117 26858729

[pcz155-B14] Emendack Y.Y., Hayes C.M., Chopra R., Sanchez J., Burow G., Xin Z., et al (2017) Early seedling growth characteristics relates to the staygreen trait and dhurrin levels in sorghum. Crop Sci. 57: 404–415.

[pcz155-B15] Forzieri G., Feyen L., Russo S., Vousdoukas M., Alfieri L., Outten S., et al (2016) Multi-hazard assessment in Europe under climate change. Clim. Change 137: 105–119.

[pcz155-B16] Frenck G., van der Linden L., Mikkelsen T.N., Brix H., J�rgensen R.B. (2011) Increased CO_2_ does not compensate for negative effects on yield caused by higher temperature and O_3_ in *Brassica napus* L. Eur. J. Agron. 35: 127–134.

[pcz155-B17] Fujita N. (2015) Manipulation of rice starch properties for application *In* Starch. Edited by Nakamura, Y. pp. 335–369. Springer, Tokyo.

[pcz155-B18] Fujita N., Yoshida M., Asakura N., Ohdan T., Miyao A., Hirochika H., et al (2006) Function and characterization of starch synthase I using mutants in rice. Plant Physiol. 140: 1070–1084. 16443699 10.1104/pp.105.071845 PMC1400558

[pcz155-B19] Gordon A., Ryle G., Powell C., Mitchell D. (1980) Export, mobilization, and respiration of assimilates in uniculm barley during light and darkness. J. Exp. Bot. 31: 461–473.

[pcz155-B20] Goswami S., Kumar R.R., Dubey K., Singh J.P., Tiwari S., Kumar A., et al (2016) SSH analysis of endosperm transcripts and characterization of heat stress regulated expressed sequence tags in bread wheat. Front. Plant Sci. 7: 1230. 27582756 10.3389/fpls.2016.01230 PMC4988357

[pcz155-B21] Hayes C., Weers B., Thakran M., Burow G., Xin Z., Emendack Y., et al (2015) Discovery of a dhurrin QTL in Sorghum bicolor: Co-localization of dhurrin biosynthesis and a novel stay-green QTL. Crop Sci. 56: 104–112.

[pcz155-B22] Ingvordsen C.H., Backes G., Lyngkj�r M.F., Peltonen-Sainio P., Jensen J.D., Jalli M., et al (2015) Significant decrease in yield under future climate conditions: stability and production of 138 spring barley accessions. Eur. J. Agron. 63: 105–113.

[pcz155-B23] Jammer A., Gasperl A., Luschin-Ebengreuth N., Heyneke E., Chu H., Cantero-Navarro E., et al (2015) Simple and robust determination of the activity signature of key carbohydrate metabolism enzymes for physiological phenotyping in model and crop plants. J. Exp. Bot. 66: 5531–5542. 26002973 10.1093/jxb/erv228

[pcz155-B24] Jeon J.-S., Ryoo N., Hahn T.-R., Walia H., Nakamura Y. (2010) Starch biosynthesis in cereal endosperm. Plant Physiol. Biochem. 48: 383–392. 20400324 10.1016/j.plaphy.2010.03.006

[pcz155-B25] Jiang S.-S., Liang X.-N., Li X., Wang S.-L., Lv D.-W., Ma C.-Y., et al (2012) Wheat drought-responsive grain proteome analysis by linear and nonlinear 2-DE and MALDI-TOF mass spectrometry. Int. J. Mol. Sci. 13: 16065–16083. 23443111 10.3390/ijms131216065 PMC3546679

[pcz155-B26] Jin Y., Ni D.-A., Ruan Y.-L. (2009) Posttranslational elevation of cell wall invertase activity by silencing its inhibitor in tomato delays leaf senescence and increases seed weight and fruit hexose level. Plant Cell 21: 2072–2089. 19574437 10.1105/tpc.108.063719 PMC2729613

[pcz155-B27] Kalinga D.N., Bertoft E., Tetlow I., Liu Q., Yada R.Y., Seetharaman K. (2014) Evolution of amylopectin structure in developing wheat endosperm starch. Carbohydr. Polym. 112: 316–324. 25129750 10.1016/j.carbpol.2014.05.008

[pcz155-B28] Kaneko K., Sasaki M., Kuribayashi N., Suzuki H., Sasuga Y., Shiraya T., et al (2016) Proteomic and glycomic characterization of rice chalky grains produced under moderate and high-temperature conditions in field system. Rice 9: 1–16. 27246013 10.1186/s12284-016-0100-y PMC4887401

[pcz155-B29] Kingston-Smith A.H., Walker R.P., Pollock C.J. (1999) Invertase in leaves: conundrum or control point? J. Exp. Bot. 50: 735–743.

[pcz155-B30] Knoch E., Motawie M.S., Olsen C.E., M�ller B.L., Lyngkjaer M.F. (2016) Biosynthesis of the leucine derived alpha, beta-and gamma-hydroxynitrile glucosides in barley (*Hordeum vulgare* L.). Plant J. 88: 247–256. 27337134 10.1111/tpj.13247

[pcz155-B31] K�llman A., Bertoft E., Koch K., Sun C., �man P., Andersson R. (2015) Starch structure in developing barley endosperm. Int. J. Biol. Macromol. 81: 730–735. 26361866 10.1016/j.ijbiomac.2015.09.013

[pcz155-B32] Li B., Liu H., Zhang Y., Kang T., Zhang L., Tong J., et al (2013) Constitutive expression of cell wall invertase genes increases grain yield and starch content in maize. Plant Biotechnol. J. 11: 1080–1091. 23926950 10.1111/pbi.12102

[pcz155-B33] Liao J.-L., Zhou H.-W., Zhang H.-Y., Zhong P.-A., Huang Y.-J. (2014) Comparative proteomic analysis of differentially expressed proteins in the early milky stage of rice grains during high temperature stress. J. Exp. Bot. 65: 655–671. 24376254 10.1093/jxb/ert435 PMC3904723

[pcz155-B34] Lu D., Shen X., Cai X., Yan F., Lu W., Shi Y.-C. (2014) Effects of heat stress during grain filling on the structure and thermal properties of waxy maize starch. Food Chem. 143: 313–318. 24054245 10.1016/j.foodchem.2013.07.089

[pcz155-B35] Lu D., Sun X., Yan F., Wang X., Xu R., Lu W. (2013) Effects of high temperature during grain filling under control conditions on the physicochemical properties of waxy maize flour. Carbohydr. Polym. 98: 302–310. 23987349 10.1016/j.carbpol.2013.06.005

[pcz155-B36] Lunn J.E., Feil R., Hendriks J.H., Gibon Y., Morcuende R., Osuna D., et al (2006) Sugar-induced increases in trehalose 6-phosphate are correlated with redox activation of ADPglucose pyrophosphorylase and higher rates of starch synthesis in *Arabidopsis thaliana*. Biochem. J. 397: 139–148. 16551270 10.1042/BJ20060083 PMC1479759

[pcz155-B37] Luo M., Liu J., Lee R.D., Scully B.T., Guo B. (2010) Monitoring the expression of maize genes in developing kernels under drought stress using oligo-microarray. J. Integr. Plant Biol. 52: 1059–1074. 21106005 10.1111/j.1744-7909.2010.01000.x

[pcz155-B38] Mangelsen E., Kilian J., Harter K., Jansson C., Wanke D., Sundberg E. (2011) Transcriptome analysis of high-temperature stress in developing barley caryopses: early stress responses and effects on storage compound biosynthesis. Mol. Plant 4: 97–115. 20924027 10.1093/mp/ssq058

[pcz155-B39] Moller I., S�rensen I., Bernal A.J., Blaukopf C., Lee K., �bro J., et al (2007) High-throughput mapping of cell-wall polymers within and between plants using novel microarrays. Plant J. 50: 1118–1128. 17565618 10.1111/j.1365-313X.2007.03114.x

[pcz155-B40] Morell M.K., Kosar-Hashemi B., Cmiel M., Samuel M.S., Chandler P., Rahman S., et al (2003) Barley sex6 mutants lack starch synthase IIa activity and contain a starch with novel properties. Plant J. 34: 173–185. 12694593 10.1046/j.1365-313x.2003.01712.x

[pcz155-B41] Myll�rinen P., Schulman A., Salovaara H., Poutanen K. (1998) The effect of growth temperature on gelatinization properties of barley starch. Acta Agric. Scand. B—Plant Soil Sci. 48: 85–90.

[pcz155-B42] Nakamura Y., Sukurai A., Inaba Y., Kimura K., Iwasawa N., Nagamine T. (2002) The fine Structure of amylopectin in endosperm from Asian cultivated rice can be largely classified into two classes. Starch 54: 1–7.

[pcz155-B43] Nakamura Y., Utsumi Y., Sawada T., Aihara S., Utsumi C., Yoshida M., et al (2010) Characterization of the reactions of starch branching enzymes from rice endosperm. Plant Cell Physiol. 51: 776–794. 20305271 10.1093/pcp/pcq035

[pcz155-B44] Ohdan T., Sawada T., Nakamura Y. (2010) Effects of temperature on starch branching enzyme properties of rice. J. Appl. Glycosci. 58: 19–26.

[pcz155-B45] Pedersen H.L., Fangel J.U., McCleary B., Ruzanski C., Rydahl M.G., Ralet M.C., et al (2012) Versatile high resolution oligosaccharide microarrays for plant glycobiology and cell wall research. J. Biol. Chem. 287: 39429–39438. 22988248 10.1074/jbc.M112.396598 PMC3501085

[pcz155-B46] Radchuk V.V., Borisjuk L., Sreenivasulu N., Merx K., Mock H.-P., Rolletschek H., et al (2009) Spatiotemporal profiling of starch biosynthesis and degradation in the developing barley grain. Plant Physiol. 150: 190–204. 19321714 10.1104/pp.108.133520 PMC2675734

[pcz155-B47] Ratnayake W.S., Jackson D.S. (2008) Starch gelatinization. Adv. Food Nutr. Res. 55: 221–268. 10.1016/S1043-4526(08)00405-1 18772106

[pcz155-B48] Regina A., Bird A., Topping D., Bowden S., Freeman J., Barsby T., et al (2006) High-amylose wheat generated by RNA interference improves indices of large-bowel health in rats. Proc. Natl. Acad. Sci. USA 103: 3546–3551. 16537443 10.1073/pnas.0510737103 PMC1450120

[pcz155-B49] Regina A., Kosar-Hashemi B., Ling S., Li Z., Rahman S., Morell M. (2010) Control of starch branching in barley defined through differential RNAi suppression of starch branching enzyme IIa and IIb. J. Exp. Bot. 61: 1469–1482. 20156842 10.1093/jxb/erq011 PMC2837261

[pcz155-B50] Roitsch T., Gonz�lez M.-C. (2004) Function and regulation of plant invertases: sweet sensations. Trends Plant Sci. 9: 606–613. 15564128 10.1016/j.tplants.2004.10.009

[pcz155-B51] Ruan Y.-L. (2014) Sucrose metabolism: gateway to diverse carbon use and sugar signaling. Annu. Rev. Plant Biol. 65: 33–67. 24579990 10.1146/annurev-arplant-050213-040251

[pcz155-B52] Russo S., Dosio A., Graversen R.G., Sillmann J., Carrao H., Dunbar M.B., et al (2014) Magnitude of extreme heat waves in present climate and their projection in a warming world. J. Geophys. Res. Atmos. 119: 12500–12512.

[pcz155-B53] Satoh H., Nishi A., Yamashita K., Takemoto Y., Tanaka Y., Hosaka Y., et al (2003) Starch-branching enzyme I-deficient mutation specifically affects the structure and properties of starch in rice endosperm. Plant Physiol. 133: 1111–1121. 14526120 10.1104/pp.103.021527 PMC281607

[pcz155-B54] Schnyder H. (1993) The role of carbohydrate storage and redistribution in the source-sink relations of wheat and barley during grain filling—a review. New Phytol. 123: 233–245.

[pcz155-B55] Shaik S.S., Carciofi M., Martens H.J., Hebelstrup K.H., Blennow A. (2014) Starch bioengineering affects cereal grain germination and seedling establishment. J. Exp. Bot. 65: 2257–2270. 24642850 10.1093/jxb/eru107 PMC4036499

[pcz155-B56] Shi W., Muthurajan R., Rahman H., Selvam J., Peng S., Zou Y., et al (2013) Source-sink dynamics and proteomic reprogramming under elevated night temperature and their impact on rice yield and grain quality. New Phytol. 197: 825–837. 23252708 10.1111/nph.12088

[pcz155-B57] Sreenivasulu N., Schnurbusch T. (2012) A genetic playground for enhancing grain number in cereals. Trends Plant Sci. 17: 91–101. 22197176 10.1016/j.tplants.2011.11.003

[pcz155-B58] Streb S., Zeeman S.C. (2012) Starch metabolism in Arabidopsis. Arabidopsis Book 10: e0160. 23393426 10.1199/tab.0160 PMC3527087

[pcz155-B59] Sturm A., Tang G.-Q. (1999) The sucrose-cleaving enzymes of plants are crucial for development, growth and carbon partitioning. Trends Plant Sci 4: 401–407. 10498964 10.1016/s1360-1385(99)01470-3

[pcz155-B60] Sun Y., Jiao G., Liu Z., Zhang X., Li J., Guo X., et al (2017) Generation of high-amylose rice through CRISPR/Cas9-mediated targeted mutagenesis of starch branching enzymes. Front. Plant Sci. 8: 298. 28326091 10.3389/fpls.2017.00298 PMC5339335

[pcz155-B61] Sz�cs A., J�ger K., Jurca M.E., F�bi�n A., Bottka S., Zvara �., et al (2010) Histological and microarray analysis of the direct effect of water shortage alone or combined with heat on early grain development in wheat (*Triticum aestivum*). Physiol. Plant. 140: 174–188. 20573045 10.1111/j.1399-3054.2010.01394.x

[pcz155-B62] Tanamachi K., Miyazaki M., Matsuo K., Suriyasak C., Tamada A., Matsuyama K., et al (2016) Differential responses to high temperature during maturation in heat-stress-tolerant cultivars of Japonica rice. Plant Prod. Sci. 19: 300–308.

[pcz155-B63] Tester M., Langridge P. (2010) Breeding technologies to increase crop production in a changing world. Science 327: 818–822. 20150489 10.1126/science.1183700

[pcz155-B64] Tester R., South J., Morrison W., Ellis R. (1991) The effects of ambient temperature during the grain-filling period on the composition and properties of starch from four barley genotypes. J. Cereal Sci. 13: 113–127.

[pcz155-B65] Tetlow I.J., Emes M.J. (2014) A review of starch-branching enzymes and their role in amylopectin biosynthesis. IUBMB Life 66: 546–558. 25196474 10.1002/iub.1297

[pcz155-B66] Thitisaksakul M., Jim�nez R.C., Arias M.C., Beckles D.M. (2012) Effects of environmental factors on cereal starch biosynthesis and composition. J. Cereal Sci. 56: 67–80.

[pcz155-B67] Timabud T., Yin X., Pongdontri P., Komatsu S. (2016) Gel-free/label-free proteomic analysis of developing rice grains under heat stress. J. Proteomics 133: 1–19. 26655677 10.1016/j.jprot.2015.12.003

[pcz155-B68] Umemoto T., Terashima K., Nakamura Y., Satoh H. (1999) Differences in amylopectin structure between two rice varieties in relation to the effects of temperature during grain-filling. Starch 51: 58–62.

[pcz155-B69] Vamadevan V., Bertoft E., Seetharaman K. (2013) On the importance of organization of glucan chains on thermal properties of starch. Carbohydr. Polym. 92: 1653–1659. 23399203 10.1016/j.carbpol.2012.11.003

[pcz155-B70] Wallwork M., Logue S., MacLeod L., Jenner C. (1998) Effect of high temperature during grain filling on starch synthesis in the developing barley grain. Funct. Plant Biol. 25: 173–181.

[pcz155-B71] Wang E., Wang J., Zhu X., Hao W., Wang L., Li Q., et al (2008) Control of rice grain-filling and yield by a gene with a potential signature of domestication. Nat. Genet. 40: 1370–1374. 18820698 10.1038/ng.220

[pcz155-B72] Yamakawa H., Hirose T., Kuroda M., Yamaguchi T. (2007) Comprehensive expression profiling of rice grain filling-related genes under high temperature using DNA microarray. Plant Physiol. 144: 258–277. 17384160 10.1104/pp.107.098665 PMC1913800

[pcz155-B73] Zeeman S.C., Kossmann J., Smith A.M. (2010) Starch: its metabolism, evolution, and biotechnological modification in plants. Annu. Rev. Plant Biol. 61: 209–234. 20192737 10.1146/annurev-arplant-042809-112301

[pcz155-B74] Zhang Y., Pan J., Huang X., Guo D., Lou H., Hou Z., et al (2017) Differential effects of a post-anthesis heat stress on wheat (*Triticum aestivum* L.) grain proteome determined by iTRAQ. Sci. Rep. 7: 3468. 28615669 10.1038/s41598-017-03860-0 PMC5471245

[pcz155-B75] Zhang C., Zhou L., Zhu Z., Lu H., Zhou X., Qian Y., et al (2016) Characterization of grain quality and starch fine structure of two japonica rice (*Oryza sativa*) cultivars with good sensory properties at different temperatures during the filling stage. J. Agric. Food Chem. 64: 4048–4057. 27128366 10.1021/acs.jafc.6b00083

[pcz155-B76] Zheng X., Qi J., Hui H., Lin L., Wang F. (2017) Starch accumulation in hulless barley during grain filling. Bot. Stud. 5: 30. 10.1186/s40529-017-0184-8 PMC5511127 28710720

[pcz155-B77] Zwack P.J., Rashotte A.M. (2013) Cytokinin inhibition of leaf senescence. Plant Signal. Behav. 8: e24737. 23656876 10.4161/psb.24737 PMC3908980

