# Supplementary material for: Amylopectin Chain Length Dynamics and Activity Signatures of Key Carbon Metabolic Enzymes Highlight Early Maturation as Culprit for Yield Reduction of Barley Endosperm Starch after Heat Stress
Source: Plant Cell Physiol. 2019 Aug 9;60(12):2692–706. doi: 10.1093/pcp/pcz155 (PMC6896705; doi:10.1093/pcp/pcz155)
Supplement: pcz155_Supplementary_Figures-Tables [file pcz155_supplementary_figures-tables.zip › pcz155-suppl_data/Figure S3.docx]

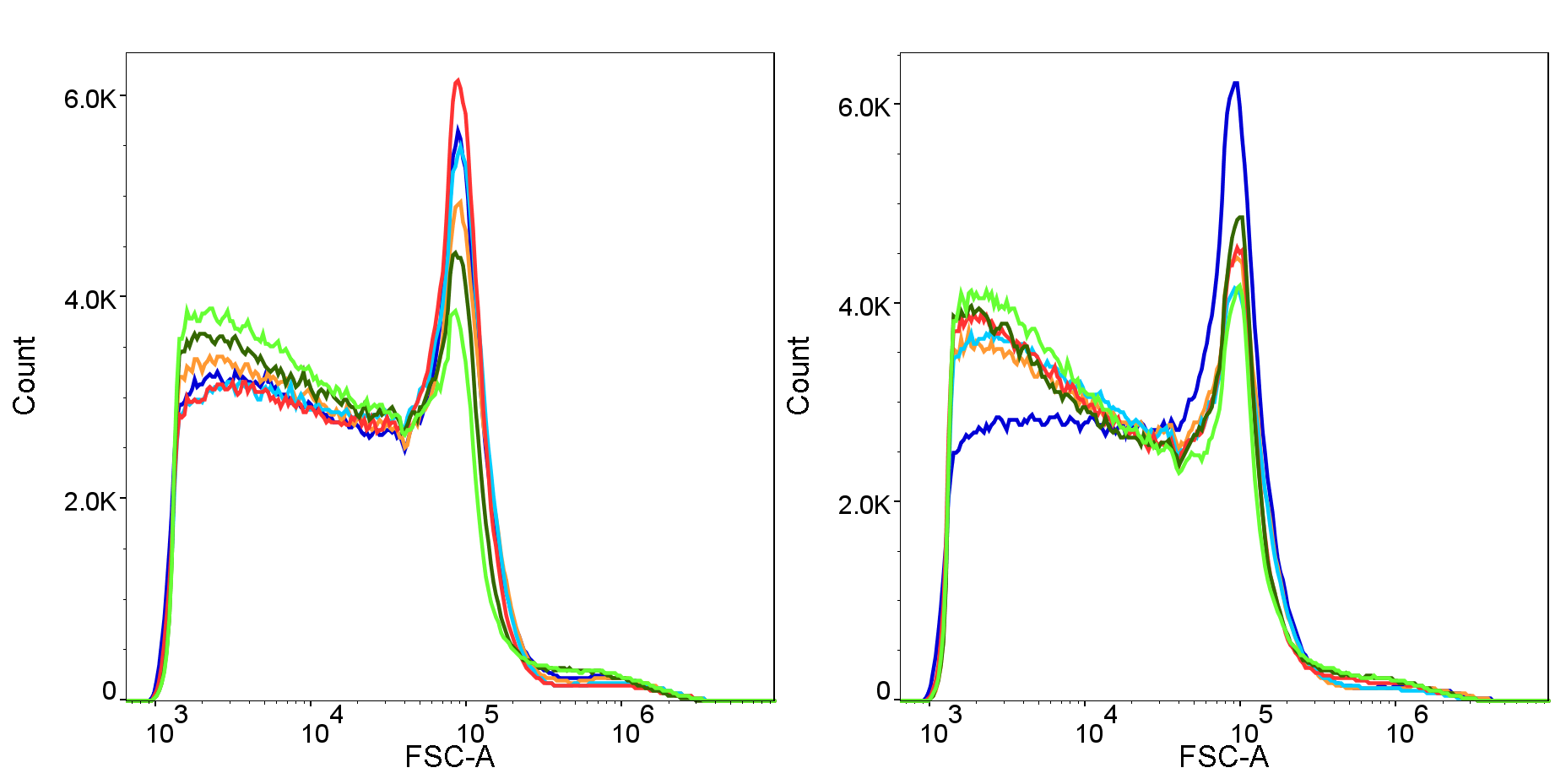


<1µm

<1µm

>8µm

>8µm

3-6µm

3-6µm

A

B

21

24

29

34

39

EP

21

24

29

34

39

EP

**Figure S 3 Flow cytometry to determine A and B starch granule count.** Forward scatter (FSC-A) histograms used to determine A and B starch granule content in plants grown under HT [A] and control [B] conditions sampled at different time points (coloured graphs represent samples taken at 21, 24, 29, 34, 39 DAF & maturity). Per sample, 50.000 granules were counted. The histograms of three biological replicates per time point (each measured in three technical replicates) were combined resulting in 450.000 counts. The FSC-A signal was converted into starch granule diameter based on a standard curve obtained using polymethyl methacrylate (PMMA) sizing microbeads (1µm, 4µm, 8µm, 20µm, 30µm). The refractive index of PMMA is 1.49, which is comparable to that of moist starch granules with 1.52 at λ=488nm. The size gates (<1µm, 3-6µm and >8µm) were selected based on the standardisation and control data.
