# Supplementary material for: Amylopectin Chain Length Dynamics and Activity Signatures of Key Carbon Metabolic Enzymes Highlight Early Maturation as Culprit for Yield Reduction of Barley Endosperm Starch after Heat Stress
Source: Plant Cell Physiol. 2019 Aug 9;60(12):2692–706. doi: 10.1093/pcp/pcz155 (PMC6896705; doi:10.1093/pcp/pcz155)
Supplement: pcz155_Supplementary_Figures-Tables [file pcz155_supplementary_figures-tables.zip › pcz155-suppl_data/Figure S1.pdf]

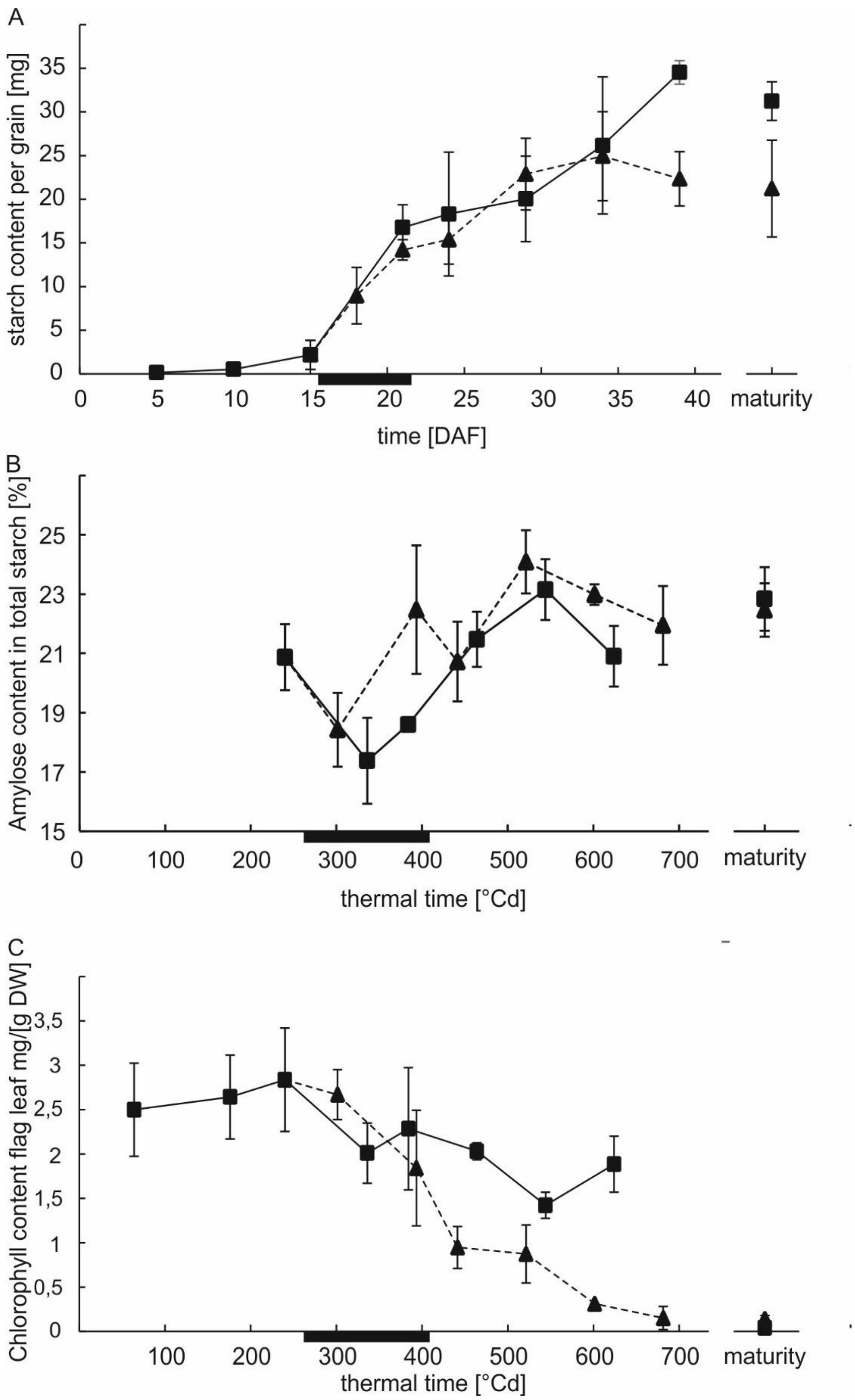
A
starch content per grain [mg]
time [DAF]
maturity
B
Amylose content in total starch [%]
thermal time [°Cd]
maturity
C
Chlorophyll content flag leaf mg/[g DW]
thermal time [°Cd]
maturity

Figure S 1 Grain starch content, amylose content *in total grain starch* and chlorophyll content in flag leaves (A) Starch content per grain [mg] in subject to time measured as days after anthesis (DAF). Time is shown as days after anthesis [DAF]. Samples were taken at DAFs 5, 10, 15, 21, 24, 29, 34, and 39 as well as from mature plants. (B) Amylose content as percentage of total grain starch plotted against thermal time. (C) Chlorophyll content in flag leaves plotted against thermal time. The duration of the heat treatment is indicated by a black bar on the abscissae. Squared symbols represent data points from plants grown under control conditions, while triangles represent plants that suffered from a heat wave between days 16 to 21 after anthesis. “Maturity” to samples taken from plants that underwent complete senescence. For samples taken during development “n” equals 3, for samples taken at maturity “n” equals 5.
