# Supplementary material for: Amylopectin Chain Length Dynamics and Activity Signatures of Key Carbon Metabolic Enzymes Highlight Early Maturation as Culprit for Yield Reduction of Barley Endosperm Starch after Heat Stress
Source: Plant Cell Physiol. 2019 Aug 9;60(12):2692–706. doi: 10.1093/pcp/pcz155 (PMC6896705; doi:10.1093/pcp/pcz155)
Supplement: pcz155_Supplementary_Figures-Tables [file pcz155_supplementary_figures-tables.zip › pcz155-suppl_data/Figure S2.pdf]

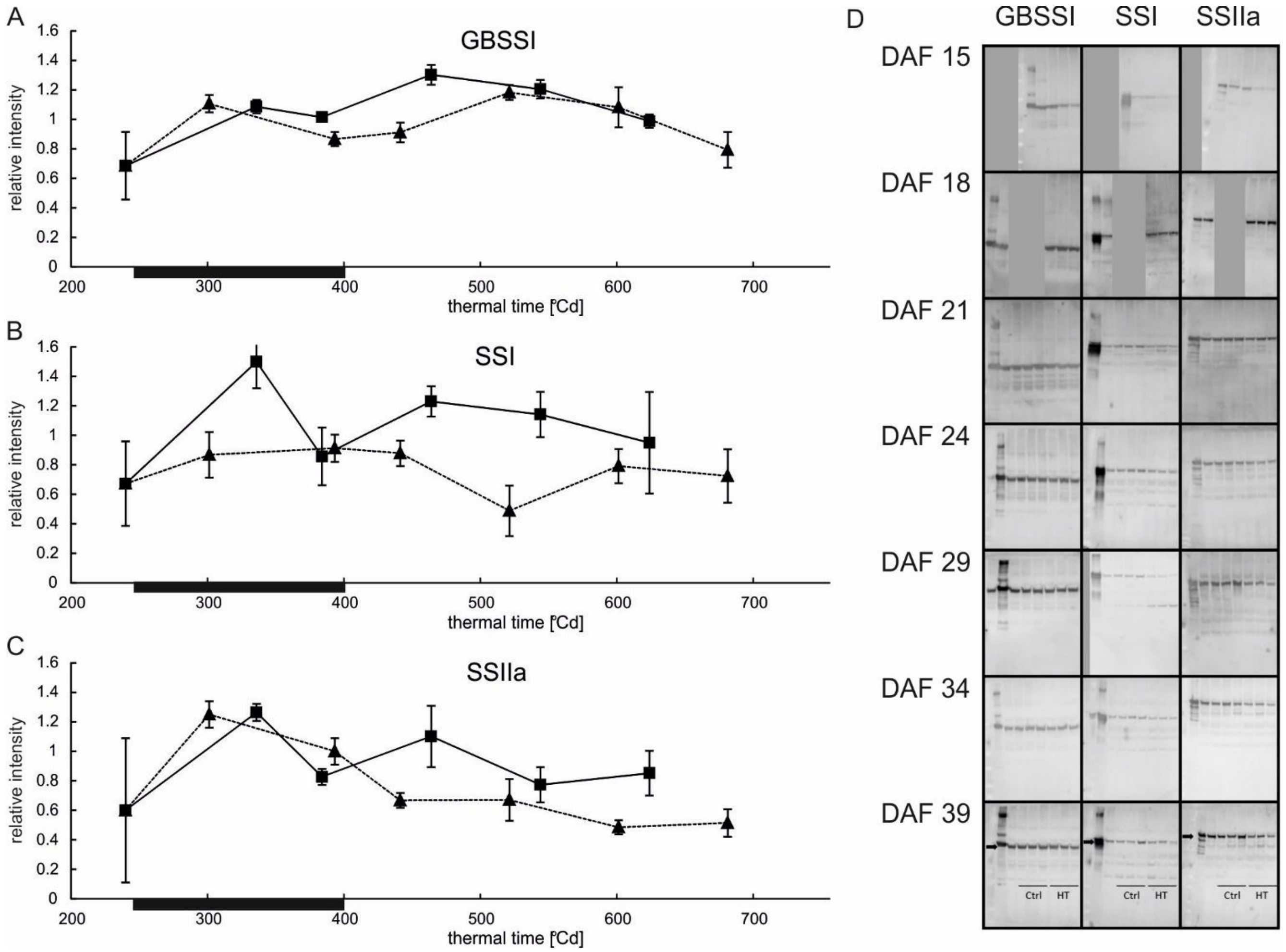

Figure S 2 Relative amounts of starch synthases over time as assessed by western blots. Relative amounts of GBSSI (A), SSI (B) and SSIIa (C) over time for control (squares) and heat treated (triangles) plants. Proteins were extracted from flour in buffer A (8 M urea, 20 mM TCEP, 2 % NP-40, 5 % PVP-25, protease inhibitor (Roche Diagnostics, Mannheim, Germany)) for 60 min at 25 °C followed by precipitation of soluble proteins in acetone for 2 h at -20 °C. Proteins were solubilized again in buffer B (8 M urea, 20 mM TCEP, protease inhibitor). Ten microgram of protein was separated in an SDS-PAGE, transferred onto a PVDF membrane and blotted against the proteins indicated. Samples from heat-treated and control plants from the same sampling day were run next to each other in the same gel. Proteins were detected using a HRP-coupled goat anti-rabbit secondary antibody (Invitrogen, ThermoFisher Scientific, Waltham, MA USA), ECL Plus reagent (ThermoFisher Scientific, Waltham, MA USA) and a Typhoon FLA 9500 laser scanner (GE Healthcare Life Sciences, Chicago, IL, USA). Band intensity was normalized against a common plant extract that was included in every gel. Error bars represent +/- the standard deviation from three individual plants. (D) Western blots used in the quantifications. The antibody used is indicated in the top, the time point in the left. In each gel, the three rightmost lanes correspond to three different HT plants and the three adjacent lanes to three different control plants, as indicated in the lower part of the DAF 39 blots. Next from the right is an extra lane with the same extract used in all gels, followed by another

lane with recombinant protein are also present in all blots. In many gels, yet another lane is visible, containing bands of negative intensity, which corresponds to molecular weight markers. Black arrows in the DAF 39 blots indicate the position of the bands that were quantified. In the case of DAF 15 plants, only the three rightmost lanes are present and relevant as the heat treatment had not yet commenced. Part of the blot that were not used for quantification in the current studies are covered by grey boxes.
