# Supplementary material for: Amylopectin Chain Length Dynamics and Activity Signatures of Key Carbon Metabolic Enzymes Highlight Early Maturation as Culprit for Yield Reduction of Barley Endosperm Starch after Heat Stress
Source: Plant Cell Physiol. 2019 Aug 9;60(12):2692–706. doi: 10.1093/pcp/pcz155 (PMC6896705; doi:10.1093/pcp/pcz155)
Supplement: pcz155_Supplementary_Figures-Tables [file pcz155_supplementary_figures-tables.zip › pcz155-suppl_data/Figure S4.pdf]

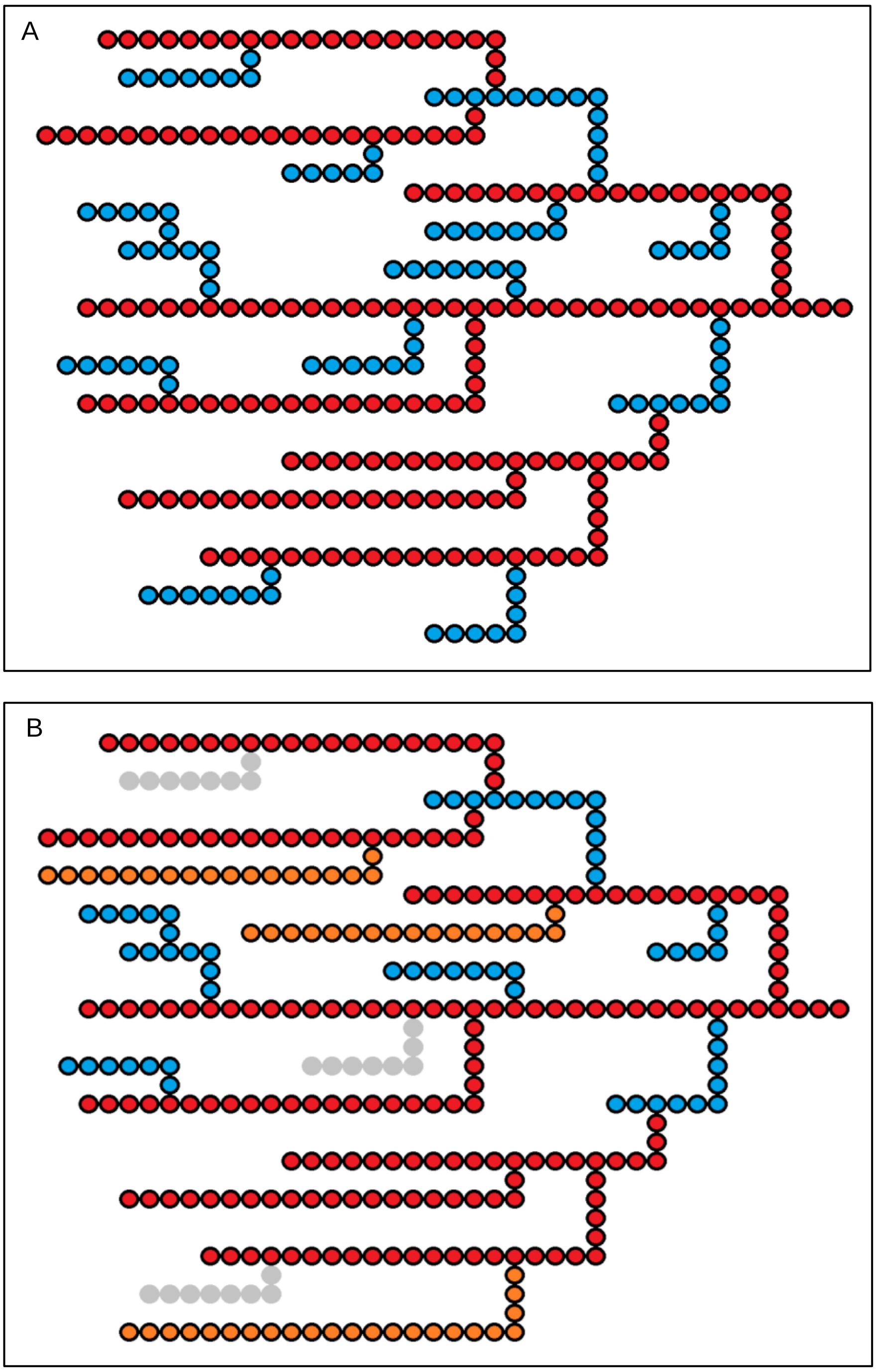

Figure S4 Schematic effects of the heat treatment on the structure of amylopectin. (A) Schematic representation of normal amylose with a certain ratio of short chains to medium and long chains. Short chains are represented in blue, medium and long chains in red. (B) After the heat treatment, two effects probably contribute to the modified chain length distribution. Some branches fail to be made (represented in light gray). This skews the balance in favor of chain elongation. At the same time, the activity of SSs that make longer chains is favored, resulting in extra elongation of short chains into medium and long chains, represented in orange. The end effect is a greater proportion of medium and long chains in amylopectin.
