# Supplementary material for: Amylopectin Chain Length Dynamics and Activity Signatures of Key Carbon Metabolic Enzymes Highlight Early Maturation as Culprit for Yield Reduction of Barley Endosperm Starch after Heat Stress
Source: Plant Cell Physiol. 2019 Aug 9;60(12):2692–706. doi: 10.1093/pcp/pcz155 (PMC6896705; doi:10.1093/pcp/pcz155)
Supplement: pcz155_Supplementary_Figures-Tables [file pcz155_supplementary_figures-tables.zip › pcz155-suppl_data/Figure S5.pdf]

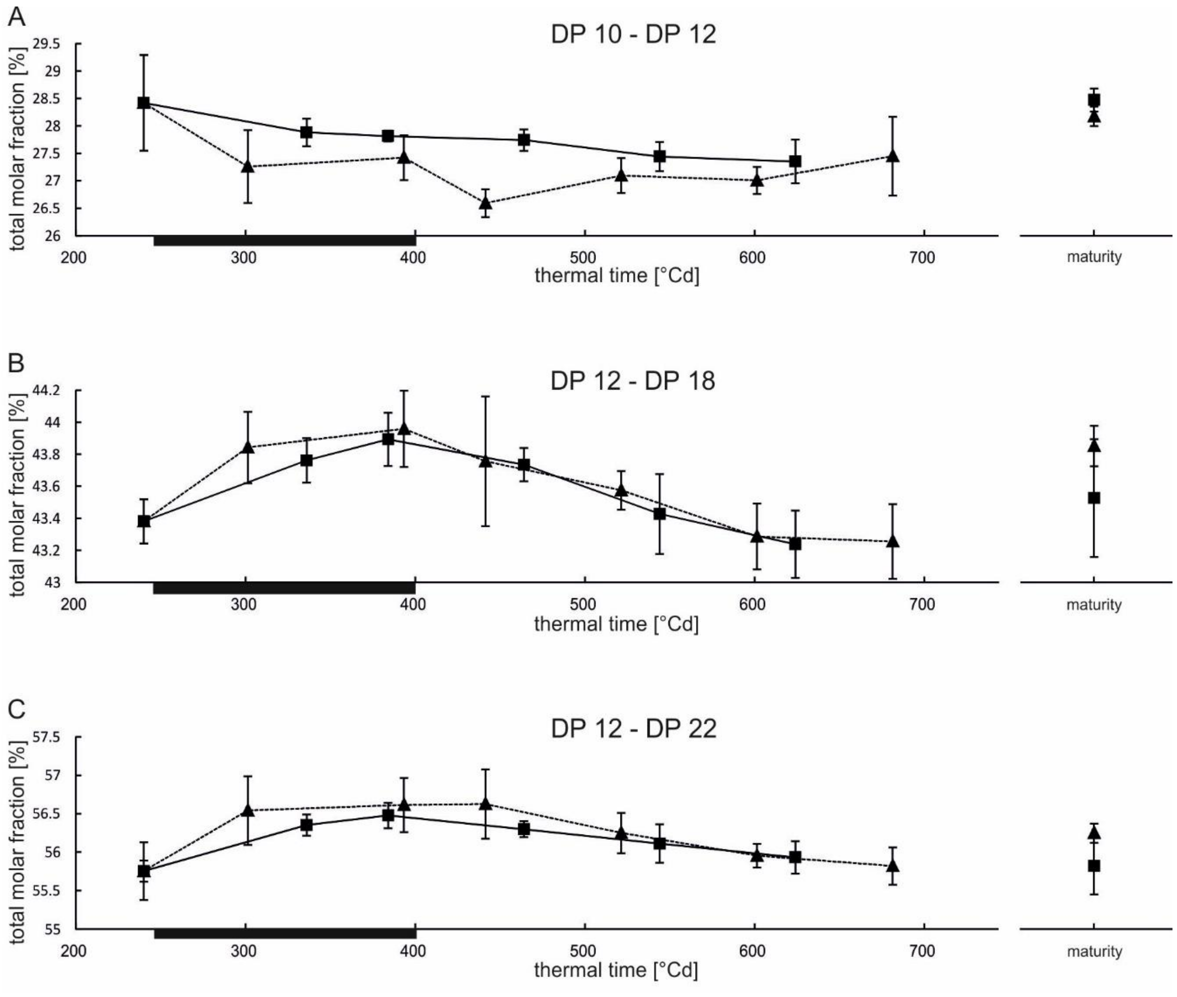

Figure S 5 Dynamics of the amylopectin chain length distribution shown as the cumulative molar fractions of DP ranges DP 8-11, DP 12-18 and DP 12-22 during grain filling in barley grown under control and heat wave conditions. Molar fractions depicted are the sum of the molar fractions of individual DPs within the DP range of DP 10-12 (A), DP 13-24 (B) and DP 31-60 (C). after normalization. The duration of the heat treatment is indicated by a black bar on the abscissae of each graph. Squared symbols represent data points from plants grown under control conditions, while triangles represent plants that suffered from a heat wave between days 16 to 21 after anthesis. Error bars represent +/- the standard deviation.
