# Supplementary material for: Amylopectin Chain Length Dynamics and Activity Signatures of Key Carbon Metabolic Enzymes Highlight Early Maturation as Culprit for Yield Reduction of Barley Endosperm Starch after Heat Stress
Source: Plant Cell Physiol. 2019 Aug 9;60(12):2692–706. doi: 10.1093/pcp/pcz155 (PMC6896705; doi:10.1093/pcp/pcz155)
Supplement: pcz155_Supplementary_Figures-Tables [file pcz155_supplementary_figures-tables.zip › pcz155-suppl_data/Figure S6.pdf]

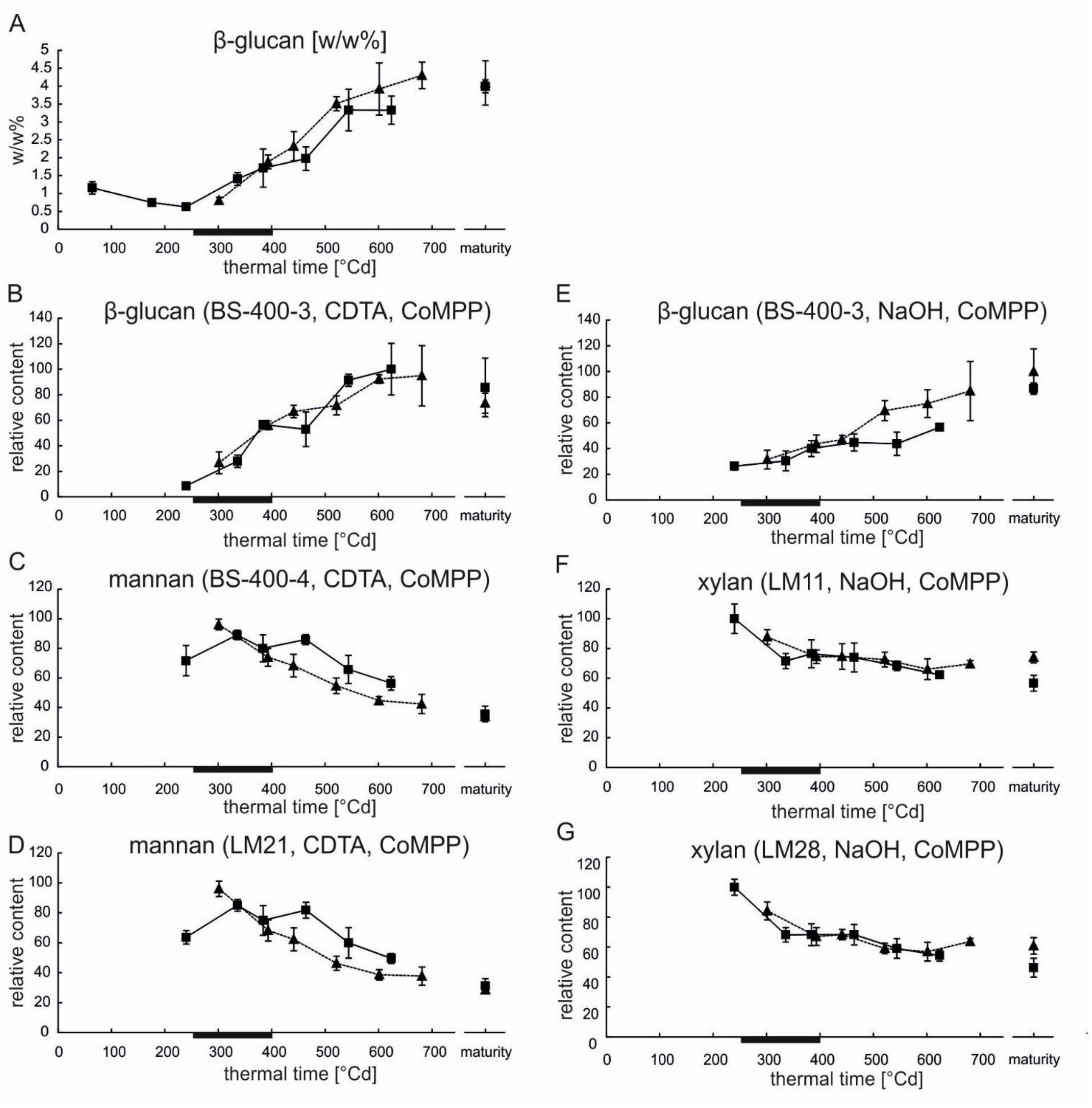

Figure S 6 Comprehensive microarray polymer profiling (CoMPP) to detect cell wall components in barley grains (A) Quantitative determination of enzyme extractable β-glucan. (B-G) Comprehensive microarray polymer

profiling (CoMPP) to detect relative abundance of cell wall components. Extractions involved a CDTA extraction step to solubilize more readily extractable components and a NaOH extraction step to solubilize more recalcitrant components. (B) β-glucan content after CDTA extraction using BS-400-3 antibody, (C) mannan content after CDTA extraction using BS-400-4 antibody, (D) mannan content after CDTA extraction using LM21 antibody, (E) β-glucan content after NaOH extraction using BS-400-3 antibody, (F) arabinoxylan content after NaOH extraction using LM11 antibody, (G) arabinoxylan content after NaOH extraction using LM28 antibody. The content of arabinoxylan was found to be different in mature grains ($p < 0.001$ and 0.005 for LM11 and LM28, respectively). Squared symbols represent data points from plants grown under control conditions, while triangles represent plants that suffered from a heat wave between days 16 to 21 after anthesis. Error bars represent +/- the standard deviation.
