# Supplementary material for: Amylopectin Chain Length Dynamics and Activity Signatures of Key Carbon Metabolic Enzymes Highlight Early Maturation as Culprit for Yield Reduction of Barley Endosperm Starch after Heat Stress
Source: Plant Cell Physiol. 2019 Aug 9;60(12):2692–706. doi: 10.1093/pcp/pcz155 (PMC6896705; doi:10.1093/pcp/pcz155)
Supplement: pcz155_Supplementary_Figures-Tables [file pcz155_supplementary_figures-tables.zip › pcz155-suppl_data/Figure S7.pdf]

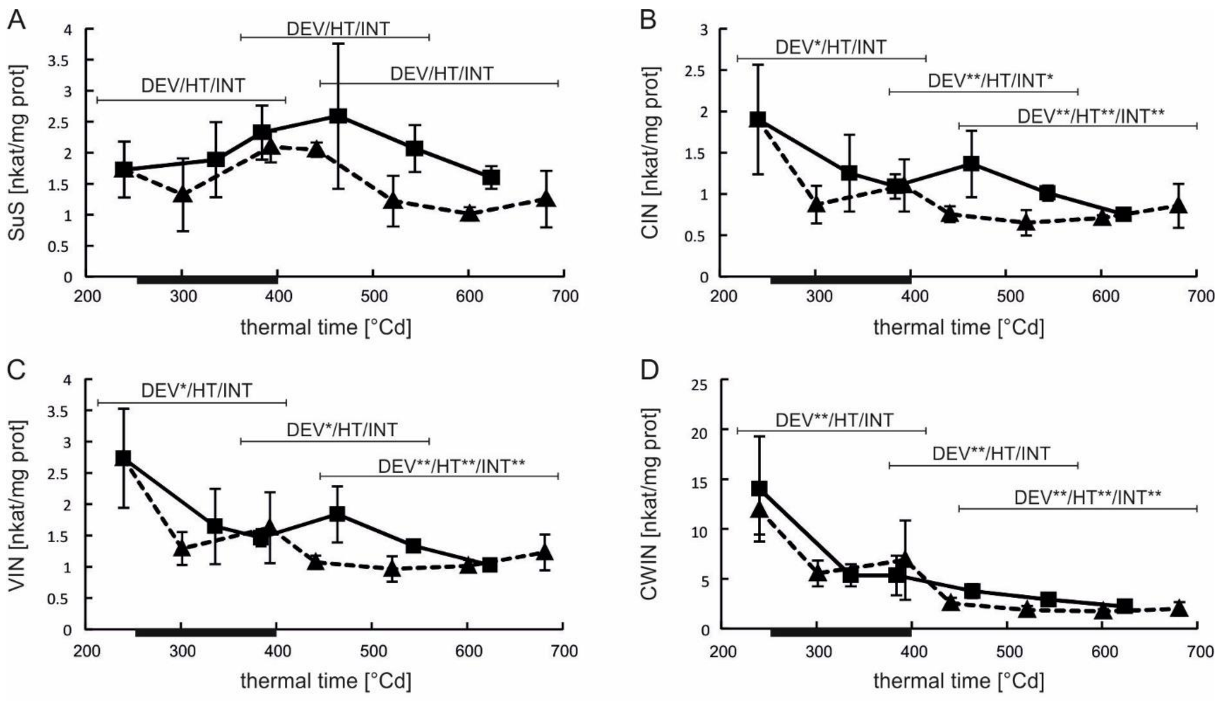

Figure S 7 Time course of the activity of sucrolytic enzymes in barley grains measured on individual plants. Depicted is the phase of starch accumulation (starting with DAF 15, 240 Cd°). The duration of the heat treatment is indicated by a black bar on the abscissae of each graph. Squared symbols represent data points from plants grown under control conditions, while triangles represent plants that suffered from a heat wave between days 16 to 21 after anthesis. Time is shown as thermal time [Cd°] to facilitate comparison between the two curves shown. Enzyme activities are shown on the left hand side in nkat per mg protein for (A) sucrose synthase (SuS), (B) cytosolic invertase (CIN), (C) vacuolar invertase (VIN) and (D) cell wall invertase (CWIN). Analysis was done on three biological replicates individually. Error bars represent +/- the standard deviation between biological replicates. The bars above the respective curves indicate ANCOVA analysis showing *=p<0.05 or **=p<0.01 significance. DEV: difference in enzyme activity over time of combined treatments; HT: difference in enzyme activity between treatments INT: interaction between changes over the indicated thermal time period and the applied treatment.
